# Schätzungen von Inzidenzen psychischer Störungen in GKV-Routinedaten: Methoden und Trends 2006 bis 2022

**DOI:** 10.1007/s00103-025-04080-y

**Published:** 2025-06-05

**Authors:** Thomas G. Grobe, Timm Frerk, Felicitas Vogelgesang, Lukas Reitzle, Frank Jacobi, Julia Thom

**Affiliations:** 1 Abteilung Gesundheitsberichterstattung und Biometrie, aQua-Institut, Maschmühlenweg 8–10, 37073 Göttingen, Deutschland; 2 Abteilung für Epidemiologie und Gesundheitsmonitoring, Robert Koch-Institut, Berlin, Deutschland; 3 Psychologische Hochschule Berlin, Berlin, Deutschland

**Keywords:** Administrative Inzidenz, Routinedaten, Psychische Störungen, Surveillance, Trends, Administrative incidence, Routine data, Mental disorders, Surveillance, Trends

## Abstract

**Einleitung:**

Routinedaten der gesetzlichen Krankenversicherung (GKV) sollen zur Surveillance nichtübertragbarer Erkrankungen (NCD) am Robert Koch-Institut (RKI) auch für die Abschätzung von administrativen Inzidenzen psychischer Störungen genutzt werden. Ziel der Studie ist es, dafür eine möglichst sensitive und praktikable Methodik zu identifizieren.

**Methoden:**

Mit ab 2005 verfügbaren Daten der BARMER-Krankenkasse, bei der ca. 10 % der Bevölkerung Deutschlands versichert waren, wurden kumulative Inzidenzen für die Jahre von 2006 bis 2022 nach schrittweisem Ausschluss von Personen mit vorausgehender Diagnosestellung in einem bis 17 Vorjahren zu Depressionen, Angststörungen, Schizophrenie-Spektrum-Störungen sowie psychischen Störungen mit einheitlicher bevölkerungsbezogener Standardisierung nach Geschlecht, Alter und Wohnregion ermittelt.

**Ergebnisse:**

Mit Ausweitung des Vorbeobachtungszeitraums lässt sich in verschiedenen Beobachtungsjahren für alle 4 Diagnosegruppen jeweils ein gleichartiger relativer Rückgang der Inzidenzschätzer feststellen. Ab einer Vorbeobachtung von 2 Jahren unterscheiden sich bei weiterer Ausdehnung der Vorbeobachtung zwar die Werte der Inzidenzschätzer, relative Veränderungen und damit Trends werden jedoch annähernd identisch abgebildet. Ein Großteil der als inzident identifizierten Personen weist in den Vorjahren bereits Diagnosen anderer psychischer Störungen auf.

**Diskussion:**

Für die Schätzung administrativer Inzidenzen erscheint der Ausschluss von Personen mit vorausgehender Diagnose in 2 Vorjahren ausreichend, um Trends abzubilden. Vergleiche und Interpretationen von Inzidenzschätzern sollten stets nur bei gleichartiger Vorbeobachtung und Methodik erfolgen. Weitere methodische Aspekte sowie Ergebnisse zu den 4 Diagnosegruppen werden diskutiert.

**Zusatzmaterial online:**

Zusätzliche Informationen sind in der Online-Version dieses Artikels (10.1007/s00103-025-04080-y) enthalten.

## Abstract (en)

**Introduction:**

Routine data from statutory health insurance (SHI) are to be used for the surveillance of non-communicable diseases (NCDs) at the Robert Koch Institute (RKI), including for the estimation of administrative incidences of mental disorders. The aim of the study is to identify a methodology that is as sensitive and practicable as possible.

**Methods:**

Using data available from 2005 onwards from the BARMER health insurance fund, with which approximately 10% of the German population was insured, cumulative incidences were determined for the years 2006 to 2022 after gradual exclusion of persons with previous diagnoses in 1 to 17 previous years for depression, anxiety disorders, schizophrenia spectrum disorders, and mental disorders with uniform population-based standardization by gender, age, and region of residence.

**Results:**

With an extension of the prior observation period, a similar relative decline in the incidence estimates can be observed for all four diagnostic groups in different observation years. From a prior observation period of two years, the values of the incidence estimators differ with further extension of the prior observation period, but relative changes and thus trends are depicted almost identically. A large proportion of the persons identified as incident already had diagnoses of other mental disorders in previous years.

**Discussion:**

For the estimation of administrative incidence rates, the exclusion of persons with a previous diagnosis in two previous years appears to be sufficient to depict trends. Comparisons and interpretations of incidence estimates should only ever be made with similar prior observation and methodology. Further methodological aspects and results for the four diagnosis groups are discussed.

## Einleitung

In Deutschland bieten die Routinedaten der gesetzlichen Krankenversicherung (GKV) mit den enthaltenen Diagnoseangaben vielfältige Möglichkeiten zur Abschätzung von Erkrankungshäufigkeiten. Routinedaten zu GKV-Versicherten stehen bereits länger mit gewissen Einschränkungen auch kassenübergreifend zur Verfügung, absehbar werden sich Zugriffsmöglichkeiten auf entsprechenden Daten im Zuge der Etablierung des Forschungsdatenzentrums Gesundheit (FDZ) voraussichtlich noch deutlich verbessern (https://www.forschungsdatenzentrum-gesundheit.de/).

### Schätzung administrativer Inzidenz zur Surveillance nichtübertragbarer Erkrankungen

Potenziale dieser Daten sollen im Rahmen einer Surveillance nichtübertragbarer Erkrankungen (NCD-Surveillance) am Robert Koch-Institut (RKI) genutzt werden. Diese wird aktuell aufbauend auf der Mental Health Surveillance [1] und Diabetes-Surveillance [2] etabliert. Ziele sind die kontinuierliche und systematische Erhebung, Analyse und Berichterstattung zur Verbreitung und Entwicklung relevanter nichtübertragbarer Erkrankungen und deren Einflussfaktoren. Neben den in den Gesundheitsstudien des RKI erhobenen Survey-Daten stellen GKV-Routinedaten zu diesem Zweck eine wichtige Ergänzung dar, u. a. da diese oft zeitnah zur Verfügung stehen und mit weniger Ressourcenaufwand analysiert werden können. Hierbei sollen neben administrativen Prävalenzen auch Inzidenzen ermittelt werden.

Inzidenzen als Maß für die Häufigkeit von Neuerkrankungen sind dabei insbesondere dann von Interesse, wenn Aussagen über Einflüsse auf Erkrankungshäufigkeiten interessieren, z. B. bei der Bewertung von Präventionsmaßnahmen. Für Diabetes mellitus liegen seitens des RKI bereits Konzepte zur Schätzung von Inzidenzen in GKV-Daten vor [3], Auswertungen zu weiteren chronisch verlaufenden körperlichen Erkrankungen sind in Vorbereitung [4]. Für die Surveillance psychischer Störungen auf Basis von GKV-Daten fehlt bisher ein entsprechender Ansatz. Dieser muss auch berücksichtigen, dass bei psychischen Störungen häufig episodische und rezidivierende Verläufe auftreten [5, 6] und kritisch diskutiert wird, wie valide Diagnosen psychischer Störungen in Routinedaten sind [7–9].

### Fehlender methodischer Standard zur Schätzung administrativer Inzidenz psychischer Störungen

Während Schätzungen zu Prävalenzen psychischer Störungen basierend auf GKV-Routinedaten in Deutschland häufiger publiziert und zumeist als „Diagnoseprävalenzen“ oder „administrative Prävalenzen“ bezeichnet werden [10–13], erscheint die Zahl der Studienpublikationen zu Inzidenzabschätzungen eher begrenzt, wobei auch Inzidenzschätzer – zur Abgrenzung von Schätzungen aus Primärerhebungsdaten – häufig als „Diagnoseinzidenzen“ oder „administrative Inzidenzen“ bezeichnet werden. Da nachfolgend ausschließlich Inzidenzschätzer basierend auf Routinedaten berichtet werden, wird hier i. d. R. auf den Zusatz „administrativ“ verzichtet.

In vorausgehenden Studien wurden unterschiedliche Vorgehensweisen und Berechnungswege für Inzidenzen gewählt, teils ohne die Auswahl im Detail zu begründen oder empirisch herzuleiten [14–30]. Dabei zeigt sich, dass insbesondere diagnosefreie Vorlaufzeiten [31] und im Vorlauf ausgeschlossene Diagnosen erheblichen Einfluss auf Inzidenzschätzer haben. Es existiert keine Vorgehensweise, die als etabliert gelten kann. Diese erscheint nicht zuletzt auch nach Erfahrungen im Zuge der COVID-19-Pandemie dringend erforderlich, da Auswertungen zu Inzidenzen psychischer Störungen zur Einschätzung der Krisenlage herangezogen wurden [26–29], ohne sich auf eine konsentierte Methodik beziehen zu können.

### Ziel der Studie

Vor dem geschilderten Hintergrund verfolgen die hier vorgestellten Analysen das Ziel, eine sensitive, robuste und replizierbare Methodik für eine möglichst zeitnahe Inzidenzschätzung psychischer Störungen für die NCD-Surveillance basierend auf Routinedaten zu identifizieren. Hierbei werden 3 psychische Störungen mit hoher Public-Health-Relevanz [32] exemplarisch betrachtet: Depressionen, Angststörungen und Schizophrenie-Spektrum-Störungen. Präsentiert werden darüber hinaus Ergebnisse zur Gesamtgruppe aller psychischen Störungen im Sinne des Kapitels V der Internationalen statistischen Klassifikation der Krankheiten und verwandter Gesundheitsprobleme, 10. Revision, German Modification (ICD-10). Schwerpunkt der Auswertungen bilden Analysen zur Frage, wie sich unterschiedlich lange diagnosefreie Vorbeobachtungszeiten auf Inzidenzschätzer und Ergebnisse zu Trends im Zeitverlauf auswirken. Weitere Abwägungen zur geeigneten Abgrenzung von Fällen mit erstmaligen oder erneuten Diagnosen psychischer Störungen werden vor dem Hintergrund der Ergebnisse diskutiert.

## Methoden

Für die Analysen wurde auf die GKV-Daten von rund 10 % der Bevölkerung Deutschlands aus den Jahren 2005 bis 2022 zurückgegriffen, die im Wissenschafts-Data-Warehouse (W-DWH) der BARMER in einer datenschutzkonform gesicherten Umgebung analysiert werden können. Berücksichtigt wurden Daten zu in Deutschland wohnhaften Versicherten der BARMER mit konsistenten Angaben zum Geschlecht und Alter innerhalb unterschiedlich definierter Beobachtungszeiträume und nachweislich durchgängiger Versicherung oder „natürlich“ durch Geburt oder Tod verkürzten Beobachtungszeiten.

### Diagnosedaten

Im W‑DWH standen diagnosebezogene Daten zur ambulanten ärztlichen und psychotherapeutischen Versorgung gemäß § 295 Sozialgesetzbuch Fünftes Buch (SGB V) sowie zu Behandlungen in Krankenhäusern gemäß § 301 SGB V für alle Jahre von 2005 bis 2022 zur Verfügung. Daten zu ambulanten Behandlungen in Krankenhäusern gemäß §§ 115 bis 119, 120 sowie 140 SGB V lagen aufgrund des schrittweisen Aufbaus des W‑DWH erst ab 2014 vor. Aus der ambulanten Versorgung wurden ausschließlich als gesichert gekennzeichnete Diagnosen berücksichtigt. Bei stationären Behandlungen wurden Haupt- und Nebendiagnosen, nicht jedoch Einweisungs- und Aufnahmediagnosen berücksichtigt.

### Zuordnungen zu Diagnosegruppen

Personen wurden anhand folgender Diagnoseschlüssel gemäß ICD-10 zu Diagnosegruppen zugeordnet: Depressionen (F32, F33.0–F33.3, F33.8, F33.9, F34.1); Angststörungen (F40, F41); Schizophrenie-Spektrum-Störungen (F20–F29). Zusätzlich wurden psychische Störungen insgesamt betrachtet (ICD-10-Kapitel V, alle Codes beginnend mit F). Konnte ein Diagnoseschlüssel in Daten im Beobachtungsjahr **J** mindestens einmalig ambulant oder stationär identifiziert werden (M1Q-Kriterium), galt die Person als diagnostiziert **D**.

### Schätzung von Prävalenzen und Inzidenzen

Diagnoseprävalenzen **P** im Jahr J werden als Quotient aus diagnostizierten Personen D und der Gesamtzahl der Personen ***n*** im selben Jahr berechnet.

1 $$P_{J}=\frac{D_{J}}{n_{J}}$$

Als Inzidenzschätzer **I **werden *kumulative Inzidenzen* berichtet.

2 $$I_{JV}=\frac{D_{JV}}{\left(\mathrm{n}_{J}-D_{V}\right)}$$

Erstmals im Jahr J diagnostizierte Personen D_JV_ (ohne Diagnosen in **V** Vorjahren) werden bei kumulativen Inzidenzen I_JV_ auf diejenige (Teil‑)Population (n_J_ – D_V_) bezogen, die dem Risiko erstmaliger Diagnosen ausgesetzt war – bereits in den V Vorjahren diagnostizierte Personen D_V_ bleiben auch im Nenner unberücksichtigt.

Inzidenzschätzer I_JV_ für die 4 Diagnosegruppen wurden für alle Beobachtungsjahre von J = 2006 bis J = 2022 ermittelt, wobei schrittweise jeweils Personen mit bereits dokumentierten Diagnosen in V = einem bis maximal V = 17 Vorjahren ab 2005 ausgeschlossen wurden. Für alle Beobachtungsjahre wurden zudem Diagnoseprävalenzen P_J_ gemäß Formel (1) ermittelt. Bei sämtlichen Analysen wurden stets identische Diagnoselisten bzw. Kriterien zur Abgrenzung von diagnostizierten Personen in Vorjahren und im Beobachtungsjahr verwendet.

### Diagnosen mit psychopathologischen Schnittmengen, andere F-Diagnosen

Ergänzend wurde für im Jahr J erstmalig diagnostizierte Personen D_JV_ ermittelt, welcher Anteil dieser Personen in den berücksichtigten V Vorjahren *ähnliche* Diagnosen erhalten hatte, bei denen durch überlappende Symptome psychopathologische Schnittmengen bestehen, z. B. gemäß Differenzialdiagnosen der relevanten Leitlinien [33–35], und bei welchem Anteil irgendeine *andere* Diagnose einer psychischen Störung dokumentiert war (vgl. auch Erläuterungen zu Tab. 1). So soll abgebildet werden, bei welchem Teil der inzidenten Fälle denkbar ist, dass dasselbe klinische Störungsbild vorausgehend lediglich diagnostisch anders eingeordnet und abweichend dokumentiert wurde und folglich keine neu aufgetretene Symptomatik impliziert.

**Tab. 1:** Inzidenzrelevante Kennzahlen zu Gruppen psychischer Störungen im Jahr 2022, abhängig von berücksichtigten Vorjahren zum Ausschluss bereits vorausgehend diagnostizierter Personen

	Vorjahre V mit Ausschluss von bereits vorausgehend diagnostizierten Personen					
	0	1	2	5	10	17
Kennwert	Depressionen (ICD-10: F32, F33.0–F33.3, F33.8, F33.9, F34.1)					
Erstdiagnostizierte Personen je 100.000	14.202	3.490	2.990	2.410	2.068	1.860
Personen unter Risiko absolut (in Tsd.)	83.799	71.381	69.430	65.163	60.492	56.645
Erstdiagnostizierte Personen absolut (in Tsd.)	11.901	2.491	2.076	1.571	1.251	1.054
Anteil Erstdiagnostizierte mit Schnittmengendiagnose (D) in V Vorjahren	–	16 %	19 %	25 %	30 %	32 %
Anteil Erstdiagnostizierte mit irgendeiner F‑Diagnose in V Vorjahren	–	48 %	56 %	68 %	77 %	84 %
Kennwert	Angststörungen (ICD-10: F40, F41)					
Erstdiagnostizierte Personen je 100.000	6.990	2.320	2.058	1.759	1.562	1.444
Personen unter Risiko absolut (in Tsd.)	83.799	77.273	75.707	72.375	68.168	64.038
Erstdiagnostizierte Personen absolut (in Tsd.)	5.858	1.792	1.558	1.273	1.065	925
Anteil Erstdiagnostizierte mit Schnittmengendiagnose (A) in V Vorjahren	–	36 %	41 %	48 %	53 %	56 %
Anteil Erstdiagnostizierte mit irgendeiner F‑Diagnose in V Vorjahren	–	56 %	63 %	74 %	82 %	87 %
Kennwert	Schizophrenie, schizotype und wahnhafte Störungen (ICD-10: F20–F29)					
Erstdiagnostizierte Personen je 100.000	991	160	141	120	110	102
Personen unter Risiko absolut (in Tsd.)	83.799	82.211	82.104	81.842	81.456	80.926
Erstdiagnostizierte Personen absolut (in Tsd.)	830	132	115	98	89	82
Anteil Erstdiagnostizierte mit Schnittmengendiagnose (S) in V Vorjahren	–	10 %	13 %	16 %	18 %	19 %
Anteil Erstdiagnostizierte mit irgendeiner F‑Diagnose in V Vorjahren	–	74 %	80 %	87 %	91 %	94 %
Kennwert	Psychische Störungen insgesamt (ICD-10-Kapitel V, alle Codes beginnend mit F)					
Erstdiagnostizierte Personen je 100.000	37.748	13.824	11.775	9.499	8.524	8.187
Personen unter Risiko absolut (in Tsd.)	83.799	51.855	45.768	34.700	25.249	18.630
Erstdiagnostizierte Personen absolut (in Tsd.)	31.633	7.168	5.389	3.296	2.152	1.525

Erläuterung: In der 1. Ergebniszeile zu jeder Diagnosegruppe werden in Spalte 1 Prävalenzschätzer und in folgenden Spalten Inzidenzschätzer ausgewiesen. Die 2. Ergebniszeile enthält Angaben zur Population unter Risiko (Gesamtbevölkerung abzüglich der in Vorjahren diagnostizierten Personen). In der 3. Ergebniszeile werden hochgerechnete Zahlen zu Personen mit prävalenten bzw. inzidenten Diagnosen in Deutschland berichtet. Die 4. Ergebniszeile nennt Anteile dieser Personen, bei denen innerhalb von V Vorjahren Diagnosen mit psychopathologischen Schnittmengen gemäß nachfolgenden Auflistungen D, A oder S dokumentiert waren. **D**: ICD-10 F00 bis F03, F06.3, F31, F34.8, F34.9, F38, F39, F41.2, F43.2, F53, Z73; **A**: F06.4, F2, F32, F33.0 bis F33.3, F33.8, F33.9, F42, F43.1, F43.2, F45.0, F45.2, F60; **S**: F05, F06.0, F06.2, F1x.5, F1x.7, F30.2, F31.2, F31.5, F32.3, F33.3; Codes jeweils mit Berücksichtigung aller weiter differenzierten Diagnosen und mit x als Platzhalter für beliebige gültige Ziffern (zu Diagnosebezeichnungen siehe Tabelle Z10 im Onlinematerial). Die 5. Ergebniszeile nennt Anteile, bei denen in Vorjahren irgendeine andere psychische Störung dokumentiert war. Für Ergebnisse zu weiteren Vorjahresausschlüssen siehe Tabelle Z9 im Onlinematerial

Auswertungsbasis: Daten zu Versicherten der BARMER mit ausreichenden Beobachtungszeiten zu min. 4.806.658 Versicherten (mit vollständiger Vorbeobachtung über 17 Jahre, darunter 1.040.365 ohne Diagnose einer psychischen Störung in 17 Vorjahren) bis max. 7.795.991 (Versicherte 2022 ohne obligate Vorbeobachtung); standardisiert D2022

### Standardisierung

Kennwerte wurden stratifiziert nach Geschlecht, 5‑Jahres-Altersgruppen (0, 1–4, 5–9, 10–14 Jahre usw. bis 90–119 Jahre) sowie Bundesland des Wohnorts ermittelt (640 Strata) und nachfolgend zusammengefasst in direkt standardisierter Form oder bevölkerungsbezogen hochgerechnet präsentiert, wobei zu allen Beobachtungsjahren einheitlich auf Angaben zur durchschnittlichen Bevölkerung in Deutschland im Jahr 2022 zurückgegriffen wurde ([36]; Angaben auf Grundlage des Zensus 2011, nachfolgend durch **D2022** gekennzeichnet).

## Ergebnisse

**Abb. 1:**
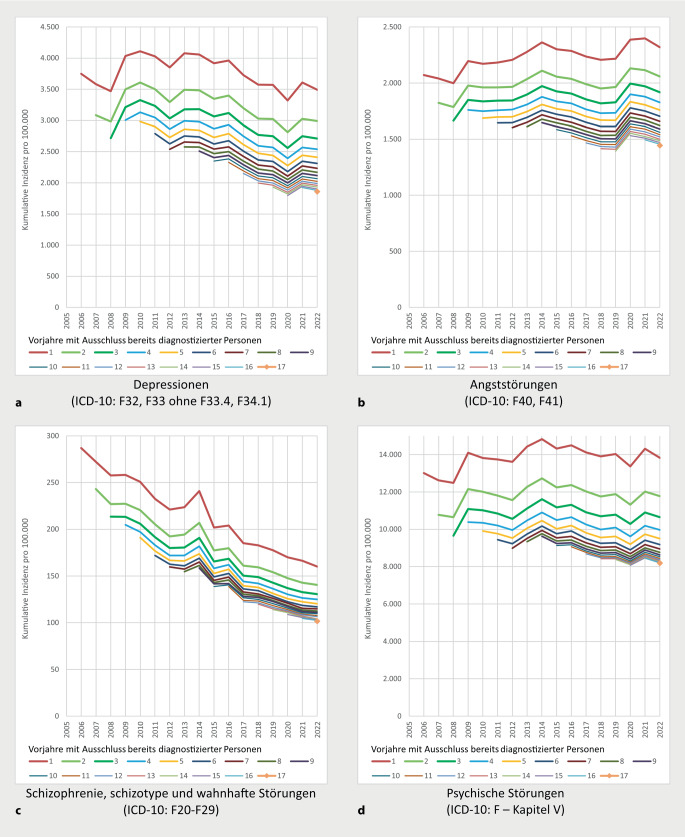
Kumulative Inzidenzen psychischer Störungen 2006 bis 2022, abhängig von berücksichtigten Vorjahren mit Ausschluss von vorausgehend diagnostizierten Personen (einheitlich standardisiert D2022, vgl. auch Tabellen Z1 bis Z4 im Onlinematerial). **a** Depressionen (ICD-10: F32, F33 ohne F33.4, F34.1), **b** Angststörungen (ICD-10: F40, F41), **c** Schizophrenie, schizotype und wahnhafte Störungen (ICD-10: F20-F29), **d** psychische Störungen (ICD-10: F – Kapitel V)

Abb. 1 zeigt kumulative Inzidenzen pro 100.000 Personen für die einzelnen Jahre von 2006 bis 2022, wobei schrittweise bereits vorausgehend Diagnostizierte aus einem bis max. 17 Vorjahren ausgeschlossen wurden. Alle der Abbildung zugrunde liegenden Zahlenangaben sind auch den Tabellen Z1 bis Z4 im Onlinematerial zu entnehmen.

### Ergebnisse zu Trends abhängig von Vorbeobachtungszeiten

Die höchsten Inzidenzschätzer zeigen sich, sofern nur ein diagnosefreies Vorjahr vorausgesetzt wird (Inzidenzschätzer je 100.000 im Jahr 2022 zu Depressionen, Angststörungen, Schizophrenie-Spektrum-Störungen sowie psychischen Störungen insgesamt: 3.490, 2.320, 160 und 13.824). Eine Ausweitung auf 2 Jahre führt bei allen Diagnosegruppen zu merklich geringeren Inzidenzschätzern (2.990, 2.058, 141 und 11.775). Mit weiter zunehmender Ausweitung des Vorbeobachtungszeitraums werden Veränderungen der Inzidenzschätzer kontinuierlich geringer. Trends der Inzidenzschätzer zeigen – abgesehen vom Niveau der Schätzwerte – bei Berücksichtigung unterschiedlicher Vorbeobachtungszeiten gleichartige Verläufe. Unterschiede hinsichtlich der relativen Veränderungen im Zeitverlauf sind bei Details in den Kurvenverläufen am ehesten bei Ausweitung der Vorbeobachtung von einem auf 2 Jahre ersichtlich.

### Veränderung der Inzidenzschätzer bei Ausweitung der Vorbeobachtung

**Abb. 2:**
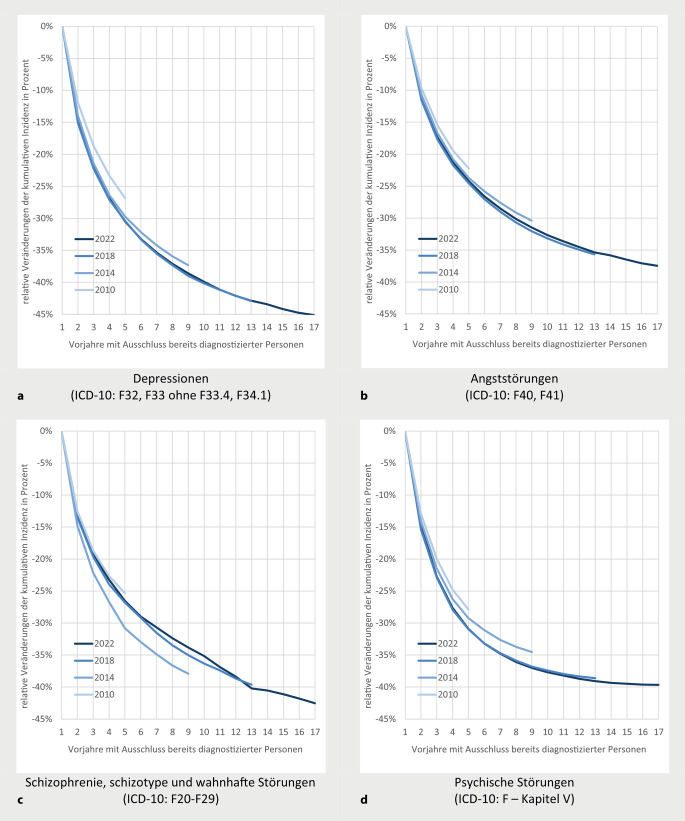
Kumulative Inzidenzen psychischer Störungen, abhängig von berücksichtigten Vorjahren mit Ausschluss von vorausgehend diagnostizierten Personen – relative Veränderungen bei Berücksichtigung von mehr als einem Vorjahr (einheitlich standardisiert D2022). **a** Depressionen (ICD-10: F32, F33 ohne F33.4, F34.1), **b** Angststörungen (ICD-10: F40, F41), **c** Schizophrenie, schizotype und wahnhafte Störungen (ICD-10: F20–F29), **d** psychische Störungen (ICD-10: F – Kapitel V)

Abb. 2 zeigt die relative Reduktion der Inzidenzschätzer zu den Jahren 2010, 2014, 2018 und 2022 bei Ausdehnung der Vorbeobachtung im Vergleich zu Schätzern bei einjähriger Vorbeobachtung. Die stärkste Reduktion bei 17-jähriger Vorbeobachtung zeigt sich bei Depressionen (−45 %), die geringste bei Angststörungen (−37 %). Keiner der Kurvenverläufe zeigt eine vollständige Abflachung. Bei allen 4 Diagnosegruppen sind nahezu identische relative Rückgänge der Inzidenzschätzer für die Jahre 2018 und 2022 zu beobachten, was auf eine Regelhaftigkeit in aktuellen Beobachtungsjahren hindeutet. Deutlich stärker ausgeprägte Abnahmen bei Inzidenzschätzern 2014 zu Diagnosen von Schizophrenie-Spektrum-Störungen resultieren maßgeblich aus der erstmaligen Berücksichtigung von Diagnosen zu ambulanten Behandlungen in Krankenhäusern ab diesem Jahr. Bei den anderen 3 Diagnosegruppen sind relative Rückgänge bei Inzidenzschätzern zu 2014 und 2010 weniger deutlich als 2018 und 2022, was vorrangig aus einer geringeren Dokumentationsdichte entsprechender Diagnosen vor 2014 resultieren dürfte.

### Inzidenzen von Depressionen in letzten Jahren niedriger als 2009 bis 2016

Schätzungen kumulativer Inzidenzen zeigen in den 4 Gruppen unterschiedliche Trends (Abb. 1). Bei Depressionen imponieren hohe kumulative Inzidenzen insbesondere in den Jahren 2009 bis 2016, gefolgt von einem deutlichen Rückgang bis 2018. Nachfolgend ergeben sich, abgesehen von einem zwischenzeitlichen Tiefstand im ersten Jahr der COVID-19-Pandemie 2020, Werte auf ähnlichem Niveau wie 2018.

### Hohe Inzidenz von Angststörungen im Jahr 2020

Anders als bei Depressionen lassen sich bei Angststörungen ab 2‑jähriger Vorbeobachtung die höchsten Inzidenzen für das Jahr 2020 ermitteln, was nachvollziehbar, jedoch in Anbetracht der zu Beginn der COVID-19-Pandemie eingeschränkten ambulanten Versorgung bemerkenswert erscheint. Hohe Inzidenzschätzer bei Angststörungen im Jahr 2014 sind mitbedingt durch die erstmalige Berücksichtigung von Diagnosen zu ambulanten Behandlungen in Krankenhäusern ab diesem Jahr, wobei sich allerdings auch bei generellem Verzicht auf eine Berücksichtigung entsprechender Diagnosen 2014 vergleichsweise hohe Schätzer zeigen (vgl. Tabelle Z5 im Onlinematerial).

### Inzidenzschätzer zu Schizophrenie-Spektrum-Störungen deutlich rückläufig

Im Hinblick auf Schizophrenien, schizotype und wahnhafte Störungen zeigt sich bei Inzidenzschätzern ein deutlicher Rückgang von 2006 bis 2022. Der Gipfel im Jahr 2014 ist auch hier durch die erstmalige Verfügbarkeit von Diagnoseangaben zu ambulanten Behandlungen in Krankenhäusern bedingt und bei genereller Nichtberücksichtigung entsprechender Diagnoseangaben nicht erkennbar (vgl. Tabelle Z6 im Onlinematerial). Ein bedeutsamer Teil der Betroffenen dürfte demnach ambulant im Krankenhaus (z. B. in Institutsambulanzen) und außerhalb der kassenärztlichen Versorgung betreut werden. Der rückläufige Trend der Inzidenzen setzt sich weitgehend unverändert auch über die ersten 3 Jahre der COVID-19-Pandemie von 2020 bis 2022 fort. Geringere relative Rückgänge (und ein noch stärker ausgeprägter Gipfel 2014) auf reduziertem Gesamtniveau lassen sich verzeichnen, sofern die Auswertungen auf die ICD-10-Diagnose F20 „Schizophrenie“ begrenzt werden (vgl. Tabelle Z7 im Onlinematerial).

### Seit 2009 moderate Veränderungen der Inzidenzen psychischer Störungen insgesamt

Kumulative Inzidenzen unter Berücksichtigung sämtlicher Diagnosen psychischer Störungen im Sinne des Kapitels V der ICD-10 bewegen sich nach einem Anstieg bis 2009 in allen Folgejahren auf ähnlichem Niveau. Zunahmen bis 2009 dürften u. a. durch den ab 2009 wirksamen morbiditätsorientierten Risikostrukturausgleich (mit Berücksichtigung von Diagnosen bei Finanzzuweisungen an Kassen) mitbedingt sein. 2014 zeigt sich ein moderat ausgeprägter Höchststand, der annähernd gleichartig auch ohne Berücksichtigung von Diagnosen zu ambulanten Behandlungen in Krankenhäusern nachweisbar ist (vgl. Tabelle Z8 im Onlinematerial). In darauffolgenden Jahren lässt sich tendenziell eher eine rückläufige Entwicklung beobachten. Nach einem zwischenzeitlichen Tiefstand im Jahr 2020 imponiert 2021 ein merklicher Anstieg, der sich 2022 jedoch nicht fortsetzt, was auf einen Nachholeffekt im Jahr 2021 hindeutet.

### Hochrechnungen auf die Bevölkerung sowie weitere Kennwerte

Tab. 1 zeigt Kennwerte zu den 4 Diagnosegruppen auch mit Hochrechnungen auf die Bevölkerung Deutschlands im Jahr 2022.

### Personen mit inzidenten Diagnosen, Personen unter Risiko sowie mit Diagnosen in Vorjahren

Ergebnisse in Tab. 1 verdeutlichen zunächst, dass die absoluten Zahlen zu inzident betroffenen Personen (jeweils 3. Ergebniszeile) mit Ausweitung der Vorbeobachtung teils merklich stärker als die kumulativen Inzidenzen zurückgehen (1. Ergebniszeile), da sich Letztere auf immer kleinere Populationen unter Risiko beziehen. 2022 war nach Auswertungen zu psychischen Störungen insgesamt bei lediglich 18,6 Mio. Personen in Deutschland noch keine F‑Diagnose in 17 Vorjahren dokumentiert, von denen dann 1,5 Mio. noch im Jahr 2022 erstmalig eine F‑Diagnose erhielten. Über den Gesamtzeitraum von 18 Jahren war damit nur bei 20,4 % der Bevölkerung bzw. 17,1 Mio. Menschen in Deutschland noch keine F‑Diagnose dokumentiert.

### Andere Diagnosen beliebiger psychischer Störungen in Vorjahren

Von Personen mit Depressionsdiagnose im Jahr 2022 und ohne eine Depressionsdiagnose im direkten Vorjahr (V = 1) wiesen 48 % in diesem Vorjahr Diagnosen anderer psychischer Störungen auf. Bei Angststörungen lag der entsprechende Anteil bei 56 %, bei Schizophrenie-Spektrum-Störungen bei 74 %. In 17 Vorjahren konnten andere F‑Diagnosen bei mehr als 80 % der Personen mit inzidenten Diagnosen 2022 in allen 3 genannten Diagnosegruppen identifiziert werden.

### Schnittmengendiagnosen in Vorjahren

Geringer fallen entsprechende Anteile bei Personen mit inzidenten Diagnosen aus, wenn in Vorjahren lediglich Diagnosen mit psychopathologischen Schnittmengen zu den primär betrachteten Diagnosegruppen identifiziert werden (vgl. jeweils 4. Ergebniszeile in Tab. 1 sowie Angaben zu berücksichtigten Codes gemäß D, A und S in der Tabellenerläuterung). So ließ sich im Jahr 2022 z. B. bei 19 % der Personen mit inzidenten Depressionsdiagnosen in 2 Vorjahren (V = 2) mindestens ein ICD-10-Code mit psychopathologischen Schnittmengen gemäß (D) identifizieren, bei 41 % der Personen mit inzidenten Angststörungsdiagnosen wurde ein Code gemäß (A) identifiziert, bei 13 % der Personen mit inzidenten Diagnosen von Schizophrenie-Spektrum-Störungen ein Code gemäß (S).

## Diskussion

Ziel der dargestellten Analysen ist es, eine optimale Methodik zur Inzidenzschätzung psychischer Störungen für die NCD-Surveillance basierend auf GKV-Routinedaten zu identifizieren. Unterschiede bei Schätzungen administrativer Inzidenzen in zuvor publizierten Studien betreffen insbesondere berücksichtigte Vorbetrachtungszeiträume, zeitliche Zuordnungen von Ereignissen sowie Abgrenzungen von inzident sowie bereits vorausgehend diagnostizierten Personen. Bei zeitlichen Zuordnungen wurden entweder Vorjahre [15–17] oder Vorquartale differenziert [14]. Aufgrund der ausgedehnten Datenverfügbarkeit wurden in den hier präsentierten Analysen lediglich Jahre unterschieden. Der zur Abgrenzung inzident diagnostizierter Personen berücksichtigte Vorbetrachtungszeitraum variiert in Studien deutlich. Es wurden sowohl ein- oder 2‑jährige [14–16] als auch diagnosefreie Zeiträume bis zu 6 Jahren vorausgesetzt [17, 18, 37]. Eine ähnliche Spannweite (9 Monate bis 10 Jahre) zeigt sich in Studien, die Inzidenzen psychischer Störungen nicht auf Basis von Routinedaten ermitteln [19–23]. Dabei scheinen Vorbeobachtungszeiten häufiger mit der Datenverfügbarkeit als inhaltlich begründet zu sein, von Gerste und Roick wird bei Depressionen ein diagnosefreier Zeitraum von 2 Jahren demgegenüber inhaltlich aus Behandlungsleitlinien hergeleitet [14, 35].

### Geringe Auswirkungen von Vorbeobachtungszeiten auf Trenddarstellungen

Mit den vorliegenden Analysen werden Ergebnisse zu Auswirkungen schrittweise erweiterter Vorbeobachtungszeiträume für effektiv 16 Beobachtungsjahre präsentiert. Inzidenzschätzer reduzieren sich demnach insbesondere bei ersten Ausweitungen der Vorbeobachtung, Unterschiede werden bei jedem folgenden Schritt kleiner.

Besonders wesentlich erscheint die Beobachtung, dass bei Ausweitung der Vorbeobachtungszeiten relative Veränderungen der Inzidenzschätzer zu aktuellen Beobachtungsjahren sehr ähnlich ausfallen (vgl. Angaben zu 2018 und 2022 in Abb. 2). Dies bildet die Grundlage für das maßgeblichste Ergebnis der Analysen: Inzidenzschätzer mit unterschiedlichen Vorbeobachtungszeiten ab 2 Jahren zeigen sehr ähnliche *relative Veränderungen* im Beobachtungsverlauf und damit auch ähnliche Trends (Abb. 1). Folglich können Inzidenzschätzer mit 2‑jähriger Vorbeobachtung und eher geringen Anforderungen an die Datenverfügbarkeit Trends im Zeitverlauf gleichermaßen und über mehr Beobachtungsjahre sichtbar machen als Schätzer mit längerer Vorbeobachtung, womit bei Trenddarstellungen Diskussionen über eine weitere Ausdehnung der Vorbeobachtung eher nachrangig erscheinen.

Die Analysen zeigen erwartungsgemäß allerdings auch, dass die Höhe von Schätzwerten administrativer Inzidenzen von berücksichtigten Vorbeobachtungszeiten abhängt, was bei Gegenüberstellungen von Schätzwerten und der Kommunikation entsprechender Ergebnisse berücksichtigt werden muss. So liegen z. B. Inzidenzschätzer zu Depressionen bei nur einjähriger Vorbeobachtung mehr als doppelt so hoch wie bei 17-jähriger Vorbeobachtung. Die mit kurzen Vorbeobachtungszeiten als inzident identifizierten Fälle sollten insofern als Personen mit Erst- *oder* erneuten Diagnosen psychischer Störungen verstanden werden.

Eine Überschätzung der Erstdiagnosen durch die Einbeziehung von erneut gestellten Diagnosen kann jedoch durch den Zweck der Schätzung gerechtfertigt sein. So sind aus Public-Health-Perspektive auch die Fälle informativ, bei denen nach einer diagnosefreien Zeit ein Rezidiv bzw. eine erneute Störungsepisode im Versorgungssystem auffällig bzw. dokumentiert wird. Auch wiederholte Episoden haben meist spezifische Auslöser und sollten im Kontext veränderter Risiko- und Schutzfaktoren psychischer Gesundheit sowie von Dokumentations- und Versorgungspraktiken diskutiert werden.

### Abgrenzungen der inzident sowie vorausgehend diagnostizierten Personen

Neben Vorbeobachtungszeiten können sich auch weitere Variationen von Vorgehensweisen merklich auf Inzidenzschätzer auswirken. (a) Im Sinne eines sog. M2Q-Kriteriums („mindestens 2 Quartale“) wird in einer Reihe von Studien bei ambulanten Diagnosen die Dokumentation in mindestens 2 von 4 konsekutiven Quartalen zur Identifikation inzident diagnostizierter Personen vorausgesetzt [14, 15]. Es wird i. d. R. aufgrund von Zweifeln an der Validität von Diagnoseangaben gewählt und führt zu geringeren Inzidenzschätzern. Während das Vorgehen bei chronischen Erkrankungen mit regelmäßigem und langfristigem medizinischen Versorgungsbedarf nachvollziehbar erscheint, ist es nicht geeignet zur Detektion kürzerer Erkrankungsepisoden, die bei psychischen Störungen nicht kategorisch auszuschließen sind. (b) In einigen Studien wurden zur Identifikation von vorausgehend betroffenen Personen auch weiter gefasste Diagnoseabgrenzungen (z. B. unter Einbeziehung von ICD-Codes mit psychopathologischen Schnittmengen) als bei der Identifikation inzidenter Fälle verwendet [z. B. 14, 30], wodurch Inzidenzschätzer gleichfalls sinken. Ähnlich wirkt sich auch aus, wenn in Studien mit Anwendung eines M2Q-Kriteriums Personen bei nur einmaliger Dokumentation der Diagnose in den Vorjahren ausgeschlossen werden [14, 15]. Begründen lassen sich derartige Vorgehensweisen mit der Annahme, dass auch einmalig dokumentierte Diagnosen sowie Diagnosen aus weiter gefassten Gruppen bereits Hinweise auf einen vorausgehenden Erkrankungsbeginn liefern. Allerdings resultiert aus einem derartigen Vorgehen das methodische Problem, dass auch offensichtlich inzidente Fälle aufgrund der Abfolgen von Diagnosedokumentationen möglicherweise niemals als inzident diagnostizierte Personen gezählt werden (z. B. wenn im Vorjahr nur „unscharfe“ Diagnosen aus weiter gefassten Gruppen und im Folgejahr eine Krankenhausbehandlung mit der eigentlichen Zieldiagnose dokumentiert sind). Von hier identifizierten Personen mit inzidenten Diagnosen von Angststörungen wären 2022 bei 2‑jähriger Vorbeobachtung mit Verweis auf Schnittmengendiagnosen in den beiden Vorjahren 41 % unberücksichtigt geblieben (Tab. 1).

Einen Ausweg zur Vervollständigung der Inzidenzzählung böte bei derartigen Konstellationen eine Berücksichtigung der jeweils vorgelagerten „unscharfen“ Schnittmengendiagnose als Inzidenzereignis, sofern nachfolgend noch Diagnosen auch nach engen Kriterien erfasst werden. Damit wären vollständige Inzidenzschätzungen jedoch erst bei Verfügbarkeit von Daten zum Folgejahr der Schnittmengendiagnose möglich. Auch die Anwendung eines M2Q-Kriteriums setzt generell Betrachtungen im Folgejahr (oder Ausnahmeregelungen) voraus, z. B. wenn eine Diagnose erstmalig im 4. Quartal eines Jahres dokumentiert ist. Inzidente Fälle am Jahresende können dann erst mit Daten zum Folgejahr verifiziert werden, was mit Verzögerungen der potenziellen Verfügbarkeit der Inzidenzschätzer verbunden ist. Insbesondere sofern Indikatoren für Neuerkrankungen sehr zeitnah bereitgestellt werden sollen, erscheint es nach den vorausgehenden Überlegungen vorteilhaft, möglichst sowohl auf ein M2Q-Kriterium zu verzichten als auch dieselben Kriterien zur Abgrenzung von inzident sowie vorausgehend diagnostizierten Personen zu verwenden.

### Ergebnisse zur Gesamtgruppe psychischer Störungen

Neben der Fokussierung auf methodische Fragen der Inzidenzschätzung ergaben die Analysen weitere durchaus bedeutsame Ergebnisse hinsichtlich der Anteile und Trends psychischer Diagnosen in der Bevölkerung. So zeigen die Auswertungen zur Gesamtgruppe psychischer Störungen, dass 2022 unter Einbeziehung von Daten ab 2005 bei nur etwa einem Fünftel der Bevölkerung (noch) keine Diagnosen psychischer Störungen dokumentiert waren. Allein in den 3 Jahren von 2020 bis 2022 waren nach standardisierten Auswertungen bei rund 52 % der Bevölkerung entsprechende Diagnosen dokumentiert (Tab. 1). F‑Diagnosen sind demnach in Deutschland insgesamt sehr häufig. Inzidenztrends dürften daher in erster Linie Hinweise auf sehr generelle Veränderungen geben können, wie z. B. eine reduzierte Dokumentation von Diagnosen zu Beginn der COVID-19-Pandemie. Zur Einschätzung des Erkrankungsgeschehens sollten besser Inzidenzschätzer zu eingegrenzten Diagnosegruppen betrachtet werden.

### Trends administrativer Inzidenzen bei näher eingegrenzten Diagnosegruppen

Die ermittelten Trends zu den 3 Diagnosegruppen zeigen merklich andere Entwicklungen als die auch zuvor regelmäßiger publizierten Diagnoseprävalenzen (vgl. auch Tabellen Z1, Z2 und Z3 im Onlinematerial). Bei steigenden Prävalenzen (z. B. bei Angststörungen) sind eher stagnierende Inzidenzen und bei stagnierenden Prävalenzen (z. B. bei Schizophrenien) dann rückläufige Inzidenzen zu beobachten (vgl. auch Prävalenzangaben in Tabellen Z1 bis Z4 im Onlinematerial). Dies deutet darauf hin, dass Diagnosen zunehmend ohne größere Unterbrechungen und über längere Zeiträume dokumentiert werden. In welcher Form dies der Fall ist und ob die Dokumentation dann auch mit einem zeitlich ausgedehnteren Therapieeinsatz einhergeht, sollten weiterführende Analysen zu einzelnen Erkrankungsgruppen klären. Dass rückläufige Trends bei administrativen Inzidenzen zu einigen psychischen Störungen auch mit kassenübergreifenden Daten nachweisbar sind, belegen umfangreiche Auswertungen mit Daten aller kassenärztlichen Vereinigungen, die im Dezember 2024 veröffentlicht wurden [30].

### Limitationen

Die Analysen beruhen auf Daten lediglich einer Krankenkasse, deren Versichertenpopulation per se nicht als repräsentativ gelten kann. Nach Standardisierung sollte dies jedoch zumindest auf methodische Studienaussagen wenig Auswirkungen haben.

Langjährig Versicherte sind eine Teilpopulation und nicht repräsentativ für alle Versicherten einer Krankenkasse, weshalb z. B. die präsentierten Abschätzungen zur Gesamtzahl der in 18 Jahren von F‑Diagnosen Betroffenen verzerrt sein könnten. Hier anderweitig nicht dargestellte *standardisierte* Prävalenzschätzer zum Jahr 2022 in dieser Teilpopulation (z. B. für psychische Störungen insgesamt: 37,40 %) unterscheiden sich jedoch kaum von Prävalenzschätzern auf Basis von Daten zu allen Versicherten im selben Jahr (37,75 %; Tab. 1 sowie Tabelle Z9 im Onlinematerial), weshalb größere Verzerrungen auch bei anderen standardisierten Ergebnissen unwahrscheinlich erscheinen.

Aspekte der Dokumentation und Validität von Diagnosen in GKV-Routinedaten, welche Inzidenzschätzer maßgeblich beeinflussen können, konnten nur kurz angesprochen werden. Dies gilt auch für die Zusammenstellungen von Diagnosen mit psychopathologischen Schnittmengen, die hier insofern nur beispielhaft betrachtet werden konnten.

Auf Darstellungen zu Trends in Subgruppen wurde verzichtet, obwohl umfangreiche Ergebnisse hierzu ermittelt wurden. Trends der administrativen Inzidenzen in Subgruppen unterscheiden sich teils erheblich von Trends in der Gesamtbevölkerung, was in Arbeiten mit erkrankungsbezogenem Fokus beachtet und betrachtet werden sollte.

## Fazit

Auf Basis der vorliegenden Ergebnisse können für Schätzungen von Inzidenzen psychischer Störungen in GKV-Routinedaten und deren Interpretation folgende Schlüsse gezogen werden:

1. Zur Detektion von Trends erscheinen bei administrativen Inzidenzen zu psychischen Störungen Ausschlüsse von Betroffenen aus 2 Vorjahren ausreichend und empfehlenswert. Weitere Ausweitungen der Vorbeobachtung verändern Trendaussagen bei hier betrachteten Diagnosegruppen kaum, verkürzen jedoch den Zeitraum, zu dem Trends berichtet werden können.
2. Schätzungen administrativer Inzidenzen hängen sehr maßgeblich von berücksichtigten Vorbeobachtungszeiten ab, die daher stets prominent dokumentiert sein sollten. Entsprechend sollten administrative Inzidenzen i. d. R. als Indikatoren für Häufigkeiten neudiagnostizierter Erkrankungen und bezogen auf relative Veränderungen interpretiert werden. Direkte Ergebnisvergleiche unterschiedlicher Studien setzen identische Vorbeobachtungszeiten und Methoden voraus.
3. In GKV-Daten identifizierte Zeitpunkte der Erstdiagnose können aus vielerlei Gründen nicht mit den Zeitpunkten eines Erkrankungsbeginns gleichgesetzt werden. Bei Erstdiagnosen psychischer Störungen finden sich häufig auch in Daten zu Vorjahren bereits Diagnosen anderer psychischer Störungen, teils auch Diagnosen mit psychopathologischen Schnittmengen, die auf einen vorausgehenden Erkrankungsbeginn hindeuten können. Viele inzidente Fälle reflektieren daher keine erstmalig dokumentierte psychische Morbidität.
4. Zur Identifikation der in Vorjahren diagnostizierten Personen sollten (dennoch) möglichst dieselben Diagnosekriterien wie zur Identifikation inzident diagnostizierter Personen verwendet werden, da andernfalls bei bestimmten Dokumentationsabfolgen auch unzweifelhaft inzidente Fälle zu keinem Zeitpunkt als solche gezählt werden.
5. Im Rahmen von NCD-Surveillance geben Trenddarstellungen zu administrativen Inzidenzen Hinweise auf veränderte Erkrankungsrisiken bzw. veränderte erkrankungsbezogene Behandlungsbedarfe im Zeitverlauf und können so als ein weiterer Baustein zu einer Einschätzung von Veränderungen der gesundheitlichen Lage beitragen, die durch Darstellungen zu administrativen Prävalenzen nicht abgebildet werden.

## Supplementary Information

Ergänzende Ergebnistabellen Z1 bis Z9 sowie Listung berücksichtigter ICD-10-Diagnosen
