# Supplementary material for: Estimations of incidences of mental disorders in statutory health insurance routine data: methodology and trends from 2006 to 2022
Source: Bundesgesundheitsblatt Gesundheitsforschung Gesundheitsschutz. 2025 Jun 5;68(11):1316–26. [Article in German] doi: 10.1007/s00103-025-04080-y (PMC12583408; doi:10.1007/s00103-025-04080-y)
Supplement: Supplementary file 1 — Ergänzende Ergebnistabellen Z1 bis Z9 sowie Listung berücksichtigter ICD-10-Diagnosen [file 103_2025_4080_MOESM1_ESM.docx]

**Onlinematerial**

Tabelle Z1: Administrative Prävalenzen sowie kumulative Inzidenzen von Depressionen in den Jahren von 2006 bis 2022 abhängig von berücksichtigten Vorjahren mit Ausschluss von bereits vorausgehend diagnostizierten Personen

|  | Prävalenz pro 100.000 | Kumulative Inzidenzen von Depressionen (ICD-10: F32, F33.0 - F33.3, F33.8, F33.9, F34.1) pro 100.000 zuvor nicht diagnostizierter Personen nach Ausschluss von Personen mit vorausgehend dokumentierten Diagnosen über 1 bis 17 Jahre | | | | | | | | | | | | | | | | |
| --- | --- | --- | --- | --- | --- | --- | --- | --- | --- | --- | --- | --- | --- | --- | --- | --- | --- | --- |
| Jahr |  | 1 | 2 | 3 | 4 | 5 | 6 | 7 | 8 | 9 | 10 | 11 | 12 | 13 | 14 | 15 | 16 | 17 |
| 2022 | 14.202 | 3.490 | 2.990 | 2.713 | 2.540 | 2.410 | 2.313 | 2.234 | 2.170 | 2.117 | 2.068 | 2.023 | 1.988 | 1.958 | 1.933 | 1.903 | 1.878 | 1.860 |
| 2021 | 14.254 | 3.607 | 3.025 | 2.749 | 2.569 | 2.443 | 2.347 | 2.270 | 2.208 | 2.152 | 2.103 | 2.061 | 2.026 | 1.995 | 1.969 | 1.946 | 1.923 |  |
| 2020 | 13.960 | 3.321 | 2.814 | 2.558 | 2.393 | 2.277 | 2.182 | 2.110 | 2.051 | 2.001 | 1.954 | 1.918 | 1.882 | 1.850 | 1.824 | 1.798 |  |  |
| 2019 | 14.153 | 3.569 | 3.022 | 2.750 | 2.569 | 2.439 | 2.344 | 2.260 | 2.190 | 2.131 | 2.083 | 2.036 | 1.998 | 1.962 | 1.932 |  |  |  |
| 2018 | 14.056 | 3.574 | 3.028 | 2.771 | 2.594 | 2.474 | 2.368 | 2.286 | 2.221 | 2.159 | 2.109 | 2.069 | 2.032 | 1.996 |  |  |  |  |
| 2017 | 14.016 | 3.728 | 3.199 | 2.925 | 2.749 | 2.616 | 2.511 | 2.425 | 2.351 | 2.291 | 2.238 | 2.193 | 2.153 |  |  |  |  |  |
| 2016 | 13.889 | 3.962 | 3.400 | 3.120 | 2.931 | 2.792 | 2.678 | 2.578 | 2.504 | 2.440 | 2.384 | 2.331 |  |  |  |  |  |  |
| 2015 | 13.479 | 3.917 | 3.347 | 3.063 | 2.869 | 2.728 | 2.626 | 2.541 | 2.470 | 2.406 | 2.351 |  |  |  |  |  |  |  |
| 2014 | 13.182 | 4.057 | 3.485 | 3.180 | 2.978 | 2.844 | 2.737 | 2.648 | 2.574 | 2.511 |  |  |  |  |  |  |  |  |
| 2013 | 12.714 | 4.078 | 3.491 | 3.178 | 2.994 | 2.860 | 2.751 | 2.655 | 2.577 |  |  |  |  |  |  |  |  |  |
| 2012 | 12.283 | 3.849 | 3.295 | 3.032 | 2.863 | 2.730 | 2.626 | 2.540 |  |  |  |  |  |  |  |  |  |  |
| 2011 | 12.131 | 4.029 | 3.501 | 3.233 | 3.045 | 2.901 | 2.791 |  |  |  |  |  |  |  |  |  |  |  |
| 2010 | 11.833 | 4.110 | 3.609 | 3.325 | 3.131 | 2.982 |  |  |  |  |  |  |  |  |  |  |  |  |
| 2009 | 11.264 | 4.035 | 3.498 | 3.211 | 3.006 |  |  |  |  |  |  |  |  |  |  |  |  |  |
| 2008 | 10.462 | 3.468 | 2.982 | 2.716 |  |  |  |  |  |  |  |  |  |  |  |  |  |  |
| 2007 | 10.343 | 3.581 | 3.086 |  |  |  |  |  |  |  |  |  |  |  |  |  |  |  |
| 2006 | 10.138 | 3.747 |  |  |  |  |  |  |  |  |  |  |  |  |  |  |  |  |

Auswertungsbasis: Daten zu Versicherten der BARMER mit ausreichenden Beobachtungszeiten variierend von n = 4.806.658 (Versicherte mit vollständiger Vorbeobachtung über 17 Jahre, darunter n = 3.066.601 ohne Diagnose in Vorjahren) bis n = 8.511.559 (Versicherte 2012, ohne erforderliche Vorbeobachtungszeiten); einheitlich standardisierte Prävalenz- und Inzidenzschätzer gemäß durchschnittlicher Bevölkerung in Deutschland 2022 nach Geschlecht, Altersgruppen und Wohnregion in Bundesländern.

Tabelle Z2: Administrative Prävalenzen sowie kumulative Inzidenzen von Angststörungen in den Jahren von 2006 bis 2022 abhängig von berücksichtigten Vorjahren mit Ausschluss von bereits vorausgehend diagnostizierten Personen

|  | Prävalenz pro 100.000 | Kumulative Inzidenzen von Angststörungen (ICD-10: F40, F41) pro 100.000 zuvor nicht diagnostizierter Personen nach Ausschluss von Personen mit vorausgehend dokumentierten Diagnosen über 1 bis 17 Jahre | | | | | | | | | | | | | | | | |
| --- | --- | --- | --- | --- | --- | --- | --- | --- | --- | --- | --- | --- | --- | --- | --- | --- | --- | --- |
| Jahr |  | 1 | 2 | 3 | 4 | 5 | 6 | 7 | 8 | 9 | 10 | 11 | 12 | 13 | 14 | 15 | 16 | 17 |
| 2022 | 6.990 | 2.320 | 2.058 | 1.918 | 1.827 | 1.759 | 1.703 | 1.658 | 1.621 | 1.590 | 1.562 | 1.540 | 1.520 | 1.500 | 1.487 | 1.470 | 1.456 | 1.444 |
| 2021 | 6.969 | 2.398 | 2.115 | 1.972 | 1.878 | 1.806 | 1.749 | 1.706 | 1.669 | 1.639 | 1.614 | 1.590 | 1.567 | 1.545 | 1.524 | 1.510 | 1.495 |  |
| 2020 | 6.766 | 2.388 | 2.132 | 1.995 | 1.899 | 1.833 | 1.778 | 1.734 | 1.695 | 1.663 | 1.636 | 1.612 | 1.590 | 1.569 | 1.553 | 1.533 |  |  |
| 2019 | 6.488 | 2.218 | 1.964 | 1.829 | 1.734 | 1.667 | 1.613 | 1.569 | 1.535 | 1.503 | 1.477 | 1.454 | 1.430 | 1.410 | 1.393 |  |  |  |
| 2018 | 6.336 | 2.206 | 1.952 | 1.819 | 1.730 | 1.669 | 1.614 | 1.571 | 1.535 | 1.505 | 1.477 | 1.454 | 1.436 | 1.417 |  |  |  |  |
| 2017 | 6.211 | 2.236 | 1.991 | 1.854 | 1.766 | 1.701 | 1.650 | 1.607 | 1.571 | 1.542 | 1.515 | 1.489 | 1.467 |  |  |  |  |  |
| 2016 | 6.097 | 2.287 | 2.038 | 1.906 | 1.818 | 1.751 | 1.697 | 1.650 | 1.616 | 1.583 | 1.557 | 1.530 |  |  |  |  |  |  |
| 2015 | 5.912 | 2.300 | 2.057 | 1.926 | 1.836 | 1.769 | 1.721 | 1.679 | 1.645 | 1.611 | 1.585 |  |  |  |  |  |  |  |
| 2014 | 5.742 | 2.363 | 2.110 | 1.972 | 1.876 | 1.809 | 1.758 | 1.716 | 1.678 | 1.647 |  |  |  |  |  |  |  |  |
| 2013 | 5.424 | 2.278 | 2.036 | 1.897 | 1.809 | 1.745 | 1.695 | 1.650 | 1.611 |  |  |  |  |  |  |  |  |  |
| 2012 | 5.211 | 2.207 | 1.967 | 1.845 | 1.764 | 1.700 | 1.647 | 1.604 |  |  |  |  |  |  |  |  |  |  |
| 2011 | 5.033 | 2.183 | 1.962 | 1.842 | 1.757 | 1.697 | 1.646 |  |  |  |  |  |  |  |  |  |  |  |
| 2010 | 4.887 | 2.172 | 1.960 | 1.837 | 1.749 | 1.687 |  |  |  |  |  |  |  |  |  |  |  |  |
| 2009 | 4.693 | 2.196 | 1.978 | 1.850 | 1.761 |  |  |  |  |  |  |  |  |  |  |  |  |  |
| 2008 | 4.322 | 1.998 | 1.787 | 1.666 |  |  |  |  |  |  |  |  |  |  |  |  |  |  |
| 2007 | 4.225 | 2.041 | 1.823 |  |  |  |  |  |  |  |  |  |  |  |  |  |  |  |
| 2006 | 4.086 | 2.072 |  |  |  |  |  |  |  |  |  |  |  |  |  |  |  |  |

Auswertungsbasis: Daten zu Versicherten der BARMER mit ausreichenden Beobachtungszeiten variierend von n = 4.806.658 (Versicherte mit vollständiger Vorbeobachtung über 17 Jahre, darunter n = 3.632.222 ohne Diagnose in Vorjahren) bis n = 8.511.559 (Versicherte 2012, ohne erforderliche Vorbeobachtungszeiten); einheitlich standardisierte Prävalenz- und Inzidenzschätzer gemäß durchschnittlicher Bevölkerung in Deutschland 2022 nach Geschlecht, Altersgruppen und Wohnregion in Bundesländern.

Tabelle Z3: Administrative Prävalenzen sowie kumulative Inzidenzen von Schizophrenien (inklusive schizotype und wahnhafte Störungen) in den Jahren von 2006 bis 2022 abhängig von berücksichtigten Vorjahren mit Ausschluss von bereits vorausgehend diagnostizierten Personen

|  | Prävalenz pro 100.000 | Kumulative Inzidenzen von Schizophrenien (Diagnosegruppe Schizophrenie, schizotype und wahnhafte Störungen, ICD-10: F20 - F29) pro 100.000 zuvor nicht diagnostizierter Personen nach Ausschluss von Personen mit vorausgehend dokumentierten Diagnosen über 1 bis 17 Jahre | | | | | | | | | | | | | | | | |
| --- | --- | --- | --- | --- | --- | --- | --- | --- | --- | --- | --- | --- | --- | --- | --- | --- | --- | --- |
| Jahr |  | 1 | 2 | 3 | 4 | 5 | 6 | 7 | 8 | 9 | 10 | 11 | 12 | 13 | 14 | 15 | 16 | 17 |
| 2022 | 991 | 160 | 141 | 130 | 125 | 120 | 117 | 115 | 112 | 111 | 110 | 108 | 106 | 104 | 104 | 103 | 102 | 102 |
| 2021 | 1.010 | 166 | 143 | 133 | 126 | 122 | 119 | 115 | 113 | 112 | 111 | 109 | 109 | 107 | 106 | 106 | 105 |  |
| 2020 | 1.013 | 170 | 148 | 137 | 130 | 126 | 122 | 121 | 118 | 117 | 116 | 114 | 113 | 112 | 111 | 109 |  |  |
| 2019 | 1.028 | 178 | 154 | 143 | 136 | 131 | 128 | 126 | 124 | 123 | 120 | 118 | 116 | 115 | 114 |  |  |  |
| 2018 | 1.034 | 183 | 159 | 149 | 142 | 138 | 134 | 131 | 129 | 127 | 125 | 123 | 121 | 120 |  |  |  |  |
| 2017 | 1.038 | 185 | 161 | 150 | 144 | 139 | 136 | 133 | 131 | 128 | 127 | 124 | 122 |  |  |  |  |  |
| 2016 | 1.054 | 204 | 180 | 168 | 162 | 157 | 153 | 149 | 146 | 142 | 141 | 139 |  |  |  |  |  |  |
| 2015 | 1.048 | 202 | 177 | 166 | 158 | 153 | 149 | 145 | 143 | 142 | 139 |  |  |  |  |  |  |  |
| 2014 | 1.055 | 241 | 207 | 191 | 182 | 174 | 169 | 165 | 161 | 159 |  |  |  |  |  |  |  |  |
| 2013 | 1.019 | 224 | 194 | 181 | 172 | 166 | 161 | 157 | 155 |  |  |  |  |  |  |  |  |  |
| 2012 | 1.018 | 221 | 192 | 180 | 172 | 167 | 163 | 160 |  |  |  |  |  |  |  |  |  |  |
| 2011 | 1.033 | 233 | 205 | 192 | 183 | 177 | 172 |  |  |  |  |  |  |  |  |  |  |  |
| 2010 | 1.058 | 251 | 220 | 206 | 197 | 191 |  |  |  |  |  |  |  |  |  |  |  |  |
| 2009 | 1.063 | 258 | 227 | 213 | 205 |  |  |  |  |  |  |  |  |  |  |  |  |  |
| 2008 | 1.050 | 258 | 227 | 214 |  |  |  |  |  |  |  |  |  |  |  |  |  |  |
| 2007 | 1.045 | 272 | 243 |  |  |  |  |  |  |  |  |  |  |  |  |  |  |  |
| 2006 | 1.025 | 287 |  |  |  |  |  |  |  |  |  |  |  |  |  |  |  |  |

Auswertungsbasis: Daten zu Versicherten der BARMER mit ausreichenden Beobachtungszeiten variierend von n = 4.806.658 (Versicherte mit vollständiger Vorbeobachtung über 17 Jahre, darunter n = 4.675.576 ohne Diagnose in Vorjahren) bis n = 8.511.559 (Versicherte 2012, ohne erforderliche Vorbeobachtungszeiten); einheitlich standardisierte Prävalenz- und Inzidenzschätzer gemäß durchschnittlicher Bevölkerung in Deutschland 2022 nach Geschlecht, Altersgruppen und Wohnregion in Bundesländern.

Tabelle Z4: Administrative Prävalenzen sowie kumulative Inzidenzen von psychischen Störungen in den Jahren von 2006 bis 2022 abhängig von berücksichtigten Vorjahren mit Ausschluss von bereits vorausgehend diagnostizierten Personen

|  | Prävalenz pro 100.000 | Kumulative Inzidenzen von psychischen Störungen (ICD-10-Kapitel V, alle Codes beginnend mit F) pro 100.000 zuvor nicht diagnostizierter Personen nach Ausschluss von Personen mit vorausgehend dokumentierten Diagnosen über 1 bis 17 Jahre | | | | | | | | | | | | | | | | |
| --- | --- | --- | --- | --- | --- | --- | --- | --- | --- | --- | --- | --- | --- | --- | --- | --- | --- | --- |
| Jahr |  | 1 | 2 | 3 | 4 | 5 | 6 | 7 | 8 | 9 | 10 | 11 | 12 | 13 | 14 | 15 | 16 | 17 |
| 2022 | 37.748 | 13.824 | 11.775 | 10.648 | 9.961 | 9.499 | 9.175 | 8.948 | 8.760 | 8.628 | 8.524 | 8.446 | 8.373 | 8.318 | 8.262 | 8.231 | 8.201 | 8.187 |
| 2021 | 37.983 | 14.310 | 12.020 | 10.895 | 10.191 | 9.730 | 9.418 | 9.184 | 9.003 | 8.882 | 8.772 | 8.689 | 8.615 | 8.556 | 8.512 | 8.473 | 8.451 |  |
| 2020 | 37.108 | 13.370 | 11.313 | 10.283 | 9.620 | 9.197 | 8.895 | 8.695 | 8.540 | 8.414 | 8.311 | 8.237 | 8.186 | 8.132 | 8.103 | 8.072 |  |  |
| 2019 | 37.493 | 14.024 | 11.881 | 10.780 | 10.091 | 9.615 | 9.280 | 9.059 | 8.886 | 8.752 | 8.641 | 8.545 | 8.484 | 8.424 | 8.387 |  |  |  |
| 2018 | 37.170 | 13.905 | 11.756 | 10.694 | 9.989 | 9.558 | 9.246 | 9.029 | 8.860 | 8.724 | 8.619 | 8.533 | 8.471 | 8.419 |  |  |  |  |
| 2017 | 36.967 | 14.118 | 12.027 | 10.912 | 10.254 | 9.814 | 9.504 | 9.273 | 9.088 | 8.952 | 8.840 | 8.758 | 8.692 |  |  |  |  |  |
| 2016 | 36.641 | 14.498 | 12.365 | 11.310 | 10.652 | 10.205 | 9.902 | 9.612 | 9.426 | 9.284 | 9.170 | 9.062 |  |  |  |  |  |  |
| 2015 | 35.902 | 14.320 | 12.237 | 11.174 | 10.489 | 10.026 | 9.759 | 9.539 | 9.376 | 9.242 | 9.130 |  |  |  |  |  |  |  |
| 2014 | 35.454 | 14.824 | 12.724 | 11.613 | 10.897 | 10.466 | 10.168 | 9.932 | 9.757 | 9.625 |  |  |  |  |  |  |  |  |
| 2013 | 34.167 | 14.426 | 12.263 | 11.118 | 10.471 | 10.049 | 9.746 | 9.513 | 9.332 |  |  |  |  |  |  |  |  |  |
| 2012 | 33.063 | 13.614 | 11.560 | 10.564 | 9.959 | 9.532 | 9.227 | 8.988 |  |  |  |  |  |  |  |  |  |  |
| 2011 | 32.705 | 13.743 | 11.799 | 10.838 | 10.204 | 9.759 | 9.438 |  |  |  |  |  |  |  |  |  |  |  |
| 2010 | 32.267 | 13.818 | 12.008 | 11.015 | 10.343 | 9.906 |  |  |  |  |  |  |  |  |  |  |  |  |
| 2009 | 31.428 | 14.096 | 12.149 | 11.084 | 10.389 |  |  |  |  |  |  |  |  |  |  |  |  |  |
| 2008 | 29.472 | 12.482 | 10.643 | 9.654 |  |  |  |  |  |  |  |  |  |  |  |  |  |  |
| 2007 | 29.228 | 12.622 | 10.775 |  |  |  |  |  |  |  |  |  |  |  |  |  |  |  |
| 2006 | 28.853 | 13.014 |  |  |  |  |  |  |  |  |  |  |  |  |  |  |  |  |

Auswertungsbasis: Daten zu Versicherten der BARMER mit ausreichenden Beobachtungszeiten variierend von n = 4.806.658 (Versicherte mit vollständiger Vorbeobachtung über 17 Jahre, darunter n = 1.040.365 ohne Diagnose in Vorjahren) bis n = 8.511.559 (Versicherte 2012, ohne erforderliche Vorbeobachtungszeiten); einheitlich standardisierte Prävalenz- und Inzidenzschätzer gemäß durchschnittlicher Bevölkerung in Deutschland 2022 nach Geschlecht, Altersgruppen und Wohnregion in Bundesländern.

Tabelle Z5: Administrative Prävalenzen sowie kumulative Inzidenzen von Angststörungen in den Jahren von 2006 bis 2022 abhängig von berücksichtigten Vorjahren mit Ausschluss von bereits vorausgehend diagnostizierten Personen – Ermittlung ohne Diagnosen zu ambulanten Behandlungen in Krankenhäusern

|  | Prävalenz pro 100.000 | Kumulative Inzidenzen von Angststörungen (ICD-10: F40, F41) pro 100.000 zuvor nicht diagnostizierter Personen nach Ausschluss von Personen mit vorausgehend dokumentierten Diagnosen über 1 bis 17 Jahre | | | | | | | | | | | | | | | | |
| --- | --- | --- | --- | --- | --- | --- | --- | --- | --- | --- | --- | --- | --- | --- | --- | --- | --- | --- |
| Jahr |  | 1 | 2 | 3 | 4 | 5 | 6 | 7 | 8 | 9 | 10 | 11 | 12 | 13 | 14 | 15 | 16 | 17 |
| 2022 | 6.881 | 2.296 | 2.036 | 1.898 | 1.807 | 1.740 | 1.685 | 1.641 | 1.604 | 1.573 | 1.545 | 1.522 | 1.502 | 1.483 | 1.470 | 1.454 | 1.439 | 1.427 |
| 2021 | 6.862 | 2.375 | 2.092 | 1.951 | 1.857 | 1.787 | 1.729 | 1.686 | 1.649 | 1.618 | 1.594 | 1.570 | 1.547 | 1.526 | 1.505 | 1.491 | 1.477 |  |
| 2020 | 6.662 | 2.363 | 2.109 | 1.973 | 1.879 | 1.812 | 1.758 | 1.714 | 1.676 | 1.643 | 1.617 | 1.593 | 1.572 | 1.551 | 1.536 | 1.515 |  |  |
| 2019 | 6.388 | 2.193 | 1.941 | 1.808 | 1.714 | 1.647 | 1.593 | 1.550 | 1.515 | 1.483 | 1.458 | 1.434 | 1.411 | 1.391 | 1.374 |  |  |  |
| 2018 | 6.244 | 2.183 | 1.930 | 1.799 | 1.711 | 1.649 | 1.593 | 1.551 | 1.515 | 1.485 | 1.457 | 1.435 | 1.416 | 1.397 |  |  |  |  |
| 2017 | 6.128 | 2.214 | 1.971 | 1.835 | 1.746 | 1.681 | 1.631 | 1.588 | 1.552 | 1.523 | 1.496 | 1.470 | 1.448 |  |  |  |  |  |
| 2016 | 6.017 | 2.267 | 2.019 | 1.886 | 1.798 | 1.730 | 1.677 | 1.630 | 1.597 | 1.564 | 1.538 | 1.511 |  |  |  |  |  |  |
| 2015 | 5.837 | 2.280 | 2.034 | 1.903 | 1.814 | 1.747 | 1.699 | 1.658 | 1.624 | 1.591 | 1.565 |  |  |  |  |  |  |  |
| 2014 | 5.671 | 2.305 | 2.061 | 1.928 | 1.835 | 1.770 | 1.720 | 1.679 | 1.642 | 1.611 |  |  |  |  |  |  |  |  |
| 2013* | 5.424 | 2.278 | 2.036 | 1.897 | 1.809 | 1.745 | 1.695 | 1.650 | 1.611 |  |  |  |  |  |  |  |  |  |
| 2012* | 5.211 | 2.207 | 1.967 | 1.845 | 1.764 | 1.700 | 1.647 | 1.604 |  |  |  |  |  |  |  |  |  |  |
| 2011* | 5.033 | 2.183 | 1.962 | 1.842 | 1.757 | 1.697 | 1.646 |  |  |  |  |  |  |  |  |  |  |  |
| 2010* | 4.887 | 2.172 | 1.960 | 1.837 | 1.749 | 1.687 |  |  |  |  |  |  |  |  |  |  |  |  |
| 2009* | 4.693 | 2.196 | 1.978 | 1.850 | 1.761 |  |  |  |  |  |  |  |  |  |  |  |  |  |
| 2008* | 4.322 | 1.998 | 1.787 | 1.666 |  |  |  |  |  |  |  |  |  |  |  |  |  |  |
| 2007* | 4.225 | 2.041 | 1.823 |  |  |  |  |  |  |  |  |  |  |  |  |  |  |  |
| 2006* | 4.086 | 2.072 |  |  |  |  |  |  |  |  |  |  |  |  |  |  |  |  |

* Jahre stets ohne Berücksichtigung von Diagnosen zu ambulanten Behandlungen in Krankenhäusern – Ergebnisse bei ergänzender Auswertung nicht verändert.
Auswertungsbasis: Daten zu Versicherten der BARMER mit ausreichenden Beobachtungszeiten variierend von n = 4.806.658 (Versicherte mit vollständiger Vorbeobachtung über 17 Jahre, darunter n = 3.637.070 ohne Diagnose in Vorjahren) bis n = 8.511.559 (Versicherte 2012, ohne erforderliche Vorbeobachtungszeiten); einheitlich standardisierte Prävalenz- und Inzidenzschätzer gemäß durchschnittlicher Bevölkerung in Deutschland 2022 nach Geschlecht, Altersgruppen und Wohnregion in Bundesländern.

Tabelle Z6: Administrative Prävalenzen sowie kumulative Inzidenzen von Schizophrenien (inklusive schizotype und wahnhafte Störungen) in den Jahren von 2006 bis 2022 abhängig von berücksichtigten Vorjahren mit Ausschluss von bereits vorausgehend diagnostizierten Personen – Ermittlung ohne Diagnosen zu ambulanten Behandlungen in Krankenhäusern

|  | Prävalenz pro 100.000 | Kumulative Inzidenzen von Schizophrenien (Diagnosegruppe Schizophrenie, schizotype und wahnhafte Störungen, ICD-10: F20 - F29) pro 100.000 zuvor nicht diagnostizierter Personen nach Ausschluss von Personen mit vorausgehend dokumentierten Diagnosen über 1 bis 17 Jahre | | | | | | | | | | | | | | | | |
| --- | --- | --- | --- | --- | --- | --- | --- | --- | --- | --- | --- | --- | --- | --- | --- | --- | --- | --- |
| Jahr |  | 1 | 2 | 3 | 4 | 5 | 6 | 7 | 8 | 9 | 10 | 11 | 12 | 13 | 14 | 15 | 16 | 17 |
| 2022 | 954 | 165 | 142 | 131 | 125 | 120 | 116 | 114 | 111 | 110 | 108 | 106 | 105 | 102 | 102 | 101 | 101 | 100 |
| 2021 | 973 | 172 | 144 | 133 | 126 | 121 | 117 | 114 | 112 | 110 | 109 | 107 | 107 | 105 | 104 | 104 | 103 |  |
| 2020 | 976 | 173 | 148 | 137 | 130 | 125 | 121 | 119 | 116 | 116 | 114 | 112 | 111 | 110 | 109 | 107 |  |  |
| 2019 | 993 | 182 | 155 | 143 | 136 | 131 | 127 | 125 | 122 | 121 | 119 | 116 | 115 | 113 | 112 |  |  |  |
| 2018 | 1.000 | 187 | 161 | 150 | 142 | 137 | 133 | 130 | 128 | 126 | 124 | 122 | 120 | 119 |  |  |  |  |
| 2017 | 1.005 | 191 | 163 | 151 | 144 | 138 | 134 | 131 | 129 | 126 | 125 | 122 | 120 |  |  |  |  |  |
| 2016 | 1.020 | 209 | 181 | 168 | 161 | 156 | 151 | 147 | 144 | 140 | 139 | 137 |  |  |  |  |  |  |
| 2015 | 1.014 | 206 | 178 | 165 | 157 | 151 | 147 | 143 | 141 | 139 | 136 |  |  |  |  |  |  |  |
| 2014 | 1.023 | 218 | 190 | 177 | 170 | 163 | 159 | 156 | 153 | 150 |  |  |  |  |  |  |  |  |
| 2013* | 1.019 | 224 | 194 | 181 | 172 | 166 | 161 | 157 | 155 |  |  |  |  |  |  |  |  |  |
| 2012* | 1.018 | 221 | 192 | 180 | 172 | 167 | 163 | 160 |  |  |  |  |  |  |  |  |  |  |
| 2011* | 1.033 | 233 | 205 | 192 | 183 | 177 | 172 |  |  |  |  |  |  |  |  |  |  |  |
| 2010* | 1.058 | 251 | 220 | 206 | 197 | 191 |  |  |  |  |  |  |  |  |  |  |  |  |
| 2009* | 1.063 | 258 | 227 | 213 | 205 |  |  |  |  |  |  |  |  |  |  |  |  |  |
| 2008* | 1.050 | 258 | 227 | 214 |  |  |  |  |  |  |  |  |  |  |  |  |  |  |
| 2007* | 1.045 | 272 | 243 |  |  |  |  |  |  |  |  |  |  |  |  |  |  |  |
| 2006* | 1.025 | 287 |  |  |  |  |  |  |  |  |  |  |  |  |  |  |  |  |

* Jahre stets ohne Berücksichtigung von Diagnosen zu ambulanten Behandlungen in Krankenhäusern – Ergebnisse bei ergänzender Auswertung nicht verändert.
Auswertungsbasis: Daten zu Versicherten der BARMER mit ausreichenden Beobachtungszeiten variierend von n = 4.806.658 (Versicherte mit vollständiger Vorbeobachtung über 17 Jahre, darunter n = 4.676.386 ohne Diagnose in Vorjahren) bis n = 8.511.559 (Versicherte 2012, ohne erforderliche Vorbeobachtungszeiten); einheitlich standardisierte Prävalenz- und Inzidenzschätzer gemäß durchschnittlicher Bevölkerung in Deutschland 2022 nach Geschlecht, Altersgruppen und Wohnregion in Bundesländern.

Tabelle Z7: Administrative Prävalenzen sowie kumulative Inzidenzen von Schizophrenien in den Jahren von 2006 bis 2022 abhängig von berücksichtigten Vorjahren mit Ausschluss von bereits vorausgehend diagnostizierten Personen

|  | Prävalenz pro 100.000 | Kumulative Inzidenzen von Schizophrenien (Schizophrenie, ICD-10: F20) pro 100.000 zuvor nicht diagnostizierter Personen nach Ausschluss von Personen mit vorausgehend dokumentierten Diagnosen über 1 bis 17 Jahre | | | | | | | | | | | | | | | | |
| --- | --- | --- | --- | --- | --- | --- | --- | --- | --- | --- | --- | --- | --- | --- | --- | --- | --- | --- |
| Jahr |  | 1 | 2 | 3 | 4 | 5 | 6 | 7 | 8 | 9 | 10 | 11 | 12 | 13 | 14 | 15 | 16 | 17 |
| 2022 | 575 | 72 | 60 | 54 | 52 | 49 | 47 | 46 | 45 | 44 | 44 | 43 | 42 | 42 | 42 | 41 | 41 | 41 |
| 2021 | 580 | 74 | 61 | 55 | 52 | 50 | 47 | 45 | 45 | 43 | 43 | 42 | 42 | 41 | 40 | 40 | 39 |  |
| 2020 | 578 | 73 | 61 | 56 | 53 | 50 | 49 | 48 | 46 | 46 | 45 | 44 | 44 | 43 | 43 | 42 |  |  |
| 2019 | 582 | 78 | 65 | 59 | 56 | 53 | 51 | 50 | 49 | 48 | 47 | 46 | 45 | 44 | 43 |  |  |  |
| 2018 | 576 | 79 | 67 | 61 | 57 | 54 | 52 | 51 | 49 | 49 | 48 | 47 | 46 | 45 |  |  |  |  |
| 2017 | 571 | 75 | 63 | 58 | 55 | 52 | 50 | 49 | 48 | 47 | 46 | 45 | 45 |  |  |  |  |  |
| 2016 | 571 | 81 | 69 | 63 | 61 | 58 | 56 | 54 | 53 | 52 | 51 | 50 |  |  |  |  |  |  |
| 2015 | 568 | 81 | 70 | 64 | 60 | 58 | 56 | 54 | 52 | 52 | 50 |  |  |  |  |  |  |  |
| 2014 | 562 | 114 | 91 | 80 | 74 | 69 | 65 | 62 | 61 | 59 |  |  |  |  |  |  |  |  |
| 2013 | 522 | 87 | 72 | 64 | 60 | 57 | 54 | 52 | 51 |  |  |  |  |  |  |  |  |  |
| 2012 | 523 | 86 | 71 | 65 | 60 | 56 | 54 | 53 |  |  |  |  |  |  |  |  |  |  |
| 2011 | 527 | 92 | 77 | 71 | 66 | 63 | 60 |  |  |  |  |  |  |  |  |  |  |  |
| 2010 | 531 | 97 | 81 | 73 | 68 | 65 |  |  |  |  |  |  |  |  |  |  |  |  |
| 2009 | 527 | 96 | 79 | 72 | 68 |  |  |  |  |  |  |  |  |  |  |  |  |  |
| 2008 | 519 | 99 | 82 | 76 |  |  |  |  |  |  |  |  |  |  |  |  |  |  |
| 2007 | 508 | 99 | 84 |  |  |  |  |  |  |  |  |  |  |  |  |  |  |  |
| 2006 | 497 | 108 |  |  |  |  |  |  |  |  |  |  |  |  |  |  |  |  |

Auswertungsbasis: Daten zu Versicherten der BARMER mit ausreichenden Beobachtungszeiten variierend von n = 4.806.658 (Versicherte mit vollständiger Vorbeobachtung über 17 Jahre, darunter n = 4.753.124 ohne Diagnose in Vorjahren) bis n = 8.511.559 (Versicherte 2012, ohne erforderliche Vorbeobachtungszeiten); einheitlich standardisierte Prävalenz- und Inzidenzschätzer gemäß durchschnittlicher Bevölkerung in Deutschland 2022 nach Geschlecht, Altersgruppen und Wohnregion in Bundesländern.

Tabelle Z8: Administrative Prävalenzen sowie kumulative Inzidenzen von psychischen Störungen in den Jahren von 2006 bis 2022 abhängig von berücksichtigten Vorjahren mit Ausschluss von bereits vorausgehend diagnostizierten Personen – Ermittlung ohne Diagnosen zu ambulanten Behandlungen in Krankenhäusern

|  | Prävalenz pro 100.000 | Kumulative Inzidenzen von psychischen Störungen (ICD-10-Kapitel V, alle Codes beginnend mit F) pro 100.000 zuvor nicht diagnostizierter Personen nach Ausschluss von Personen mit vorausgehend dokumentierten Diagnosen über 1 bis 17 Jahre | | | | | | | | | | | | | | | | |
| --- | --- | --- | --- | --- | --- | --- | --- | --- | --- | --- | --- | --- | --- | --- | --- | --- | --- | --- |
| Jahr |  | 1 | 2 | 3 | 4 | 5 | 6 | 7 | 8 | 9 | 10 | 11 | 12 | 13 | 14 | 15 | 16 | 17 |
| 2022 | 37.571 | 13.820 | 11.760 | 10.630 | 9.940 | 9.477 | 9.153 | 8.927 | 8.739 | 8.606 | 8.502 | 8.423 | 8.351 | 8.296 | 8.240 | 8.208 | 8.180 | 8.165 |
| 2021 | 37.813 | 14.309 | 12.004 | 10.876 | 10.171 | 9.710 | 9.398 | 9.164 | 8.981 | 8.860 | 8.750 | 8.667 | 8.592 | 8.533 | 8.488 | 8.450 | 8.427 |  |
| 2020 | 36.934 | 13.359 | 11.294 | 10.260 | 9.598 | 9.173 | 8.874 | 8.674 | 8.518 | 8.391 | 8.289 | 8.215 | 8.162 | 8.107 | 8.078 | 8.047 |  |  |
| 2019 | 37.334 | 14.024 | 11.868 | 10.764 | 10.075 | 9.599 | 9.262 | 9.040 | 8.865 | 8.731 | 8.620 | 8.525 | 8.464 | 8.404 | 8.369 |  |  |  |
| 2018 | 37.008 | 13.892 | 11.736 | 10.674 | 9.968 | 9.534 | 9.221 | 9.004 | 8.834 | 8.697 | 8.593 | 8.508 | 8.445 | 8.393 |  |  |  |  |
| 2017 | 36.814 | 14.107 | 12.008 | 10.893 | 10.229 | 9.789 | 9.478 | 9.247 | 9.062 | 8.927 | 8.815 | 8.732 | 8.666 |  |  |  |  |  |
| 2016 | 36.488 | 14.488 | 12.347 | 11.284 | 10.625 | 10.178 | 9.876 | 9.586 | 9.400 | 9.258 | 9.144 | 9.036 |  |  |  |  |  |  |
| 2015 | 35.750 | 14.309 | 12.207 | 11.140 | 10.455 | 9.992 | 9.727 | 9.507 | 9.344 | 9.210 | 9.099 |  |  |  |  |  |  |  |
| 2014 | 35.305 | 14.699 | 12.627 | 11.528 | 10.818 | 10.392 | 10.097 | 9.864 | 9.689 | 9.558 |  |  |  |  |  |  |  |  |
| 2013* | 34.167 | 14.426 | 12.263 | 11.118 | 10.471 | 10.049 | 9.746 | 9.513 | 9.332 |  |  |  |  |  |  |  |  |  |
| 2012* | 33.063 | 13.614 | 11.560 | 10.564 | 9.959 | 9.532 | 9.227 | 8.988 |  |  |  |  |  |  |  |  |  |  |
| 2011* | 32.705 | 13.743 | 11.799 | 10.838 | 10.204 | 9.759 | 9.438 |  |  |  |  |  |  |  |  |  |  |  |
| 2010* | 32.267 | 13.818 | 12.008 | 11.015 | 10.343 | 9.906 |  |  |  |  |  |  |  |  |  |  |  |  |
| 2009* | 31.428 | 14.096 | 12.149 | 11.084 | 10.389 |  |  |  |  |  |  |  |  |  |  |  |  |  |
| 2008* | 29.472 | 12.482 | 10.643 | 9.654 |  |  |  |  |  |  |  |  |  |  |  |  |  |  |
| 2007* | 29.228 | 12.622 | 10.775 |  |  |  |  |  |  |  |  |  |  |  |  |  |  |  |
| 2006* | 28.853 | 13.014 |  |  |  |  |  |  |  |  |  |  |  |  |  |  |  |  |

* Jahre stets ohne Berücksichtigung von Diagnosen zu ambulanten Behandlungen in Krankenhäusern – Ergebnisse bei ergänzender Auswertung nicht verändert.
Auswertungsbasis: Daten zu Versicherten der BARMER mit ausreichenden Beobachtungszeiten variierend von n = 4.806.658 (Versicherte mit vollständiger Vorbeobachtung über 17 Jahre, darunter n = 1.042.803 ohne Diagnose in Vorjahren) bis n = 8.511.559 (Versicherte 2012, ohne erforderliche Vorbeobachtungszeiten); einheitlich standardisierte Prävalenz- und Inzidenzschätzer gemäß durchschnittlicher Bevölkerung in Deutschland 2022 nach Geschlecht, Altersgruppen und Wohnregion in Bundesländern.

Tabelle Z9: Inzidenz-relevante Kennzahlen zu Gruppen psychischer Störungen 2022 abhängig von berücksichtigten Vorjahren mit Ausschluss von bereits vorausgehend diagnostizierten Personen

|  | Anzahl berücksichtigte Vorjahre V zum Ausschluss bereits vorausgehend diagnostizierter Personen | | | | | | | | | | | | | | | | | |
| --- | --- | --- | --- | --- | --- | --- | --- | --- | --- | --- | --- | --- | --- | --- | --- | --- | --- | --- |
|  | 0 | 1 | 2 | 3 | 4 | 5 | 6 | 7 | 8 | 9 | 10 | 11 | 12 | 13 | 14 | 15 | 16 | 17 |
| Versicherte mit hinreichender Beobachtung (Rohwerte, in Tsd.)* | 7.796 | 7.425 | 7.254 | 7.076 | 6.908 | 6.742 | 6.581 | 6.424 | 6.276 | 6.120 | 5.979 | 5.777 | 5.587 | 5.418 | 5.237 | 5.084 | 4.940 | 4.807 |
| Kennwert | Depressionen (ICD-10: F32, F33.0 - F33.3, F33.8, F33.9, F34.1) | | | | | | | | | | | | | | | | | |
| Erstdiagnostizierte Personen je 100.000 | 14.202 | 3.490 | 2.990 | 2.713 | 2.540 | 2.410 | 2.313 | 2.234 | 2.170 | 2.117 | 2.068 | 2.023 | 1.988 | 1.958 | 1.933 | 1.903 | 1.878 | 1.860 |
| Personen unter Risiko absolut (in Tsd.) | 83.799 | 71.381 | 69.430 | 67.782 | 66.402 | 65.163 | 64.035 | 63.040 | 62.122 | 61.284 | 60.492 | 59.758 | 59.064 | 58.471 | 58.007 | 57.538 | 57.071 | 56.645 |
| Erstdiagnostizierte Personen absolut (in Tsd.) | 11.901 | 2.491 | 2.076 | 1.839 | 1.687 | 1.571 | 1.481 | 1.409 | 1.348 | 1.298 | 1.251 | 1.209 | 1.174 | 1.145 | 1.121 | 1.095 | 1.072 | 1.054 |
| Anteil Erstdiagnostizierte mit Schnittmengendiagnose (D) in V Vorjahren | -- | 16 % | 19 % | 21 % | 23 % | 25 % | 26 % | 27 % | 28 % | 29 % | 30 % | 31 % | 31 % | 31 % | 32 % | 32 % | 32 % | 32 % |
| Anteil Erstdiagnostizierte mit irgendeiner  F-Diagnose in V Vorjahren | -- | 48 % | 56 % | 61 % | 65 % | 68 % | 70 % | 72 % | 74 % | 76 % | 77 % | 79 % | 80 % | 81 % | 82 % | 82 % | 83 % | 84 % |
| Kennwert | Angststörungen (ICD-10: F40, F41) | | | | | | | | | | | | | | | | | |
| Erstdiagnostizierte Personen je 100.000 | 6.990 | 2.320 | 2.058 | 1.918 | 1.827 | 1.759 | 1.703 | 1.658 | 1.621 | 1.590 | 1.562 | 1.540 | 1.520 | 1.500 | 1.487 | 1.470 | 1.456 | 1.444 |
| Personen unter Risiko absolut (in Tsd.) | 83.799 | 77.273 | 75.707 | 74.473 | 73.382 | 72.375 | 71.418 | 70.526 | 69.675 | 68.907 | 68.168 | 67.479 | 66.821 | 66.204 | 65.662 | 65.106 | 64.556 | 64.038 |
| Erstdiagnostizierte Personen absolut (in Tsd.) | 5.858 | 1.792 | 1.558 | 1.429 | 1.340 | 1.273 | 1.216 | 1.170 | 1.129 | 1.096 | 1.065 | 1.039 | 1.015 | 993 | 976 | 957 | 940 | 925 |
| Anteil Erstdiagnostizierte mit Schnittmengendiagnose (A) in V Vorjahren | -- | 36 % | 41 % | 44 % | 46 % | 48 % | 49 % | 51 % | 52 % | 53 % | 53 % | 54 % | 54 % | 55 % | 55 % | 55 % | 55 % | 56 % |
| Anteil Erstdiagnostizierte mit irgendeiner  F-Diagnose in V Vorjahren | -- | 56 % | 63 % | 68 % | 72 % | 74 % | 76 % | 78 % | 80 % | 81 % | 82 % | 83 % | 84 % | 85 % | 85 % | 86 % | 87 % | 87 % |
| Kennwert | Schizophrenien inklusive schizotype und wahnhafte Störungen (ICD-10: F20 - F29) | | | | | | | | | | | | | | | | | |
| Erstdiagnostizierte Personen je 100.000 | 991 | 160 | 141 | 130 | 125 | 120 | 117 | 115 | 112 | 111 | 110 | 108 | 106 | 104 | 104 | 103 | 102 | 102 |
| Personen unter Risiko absolut (in Tsd.) | 83.799 | 82.211 | 82.104 | 82.008 | 81.920 | 81.842 | 81.758 | 81.683 | 81.604 | 81.529 | 81.456 | 81.376 | 81.288 | 81.205 | 81.137 | 81.067 | 80.995 | 80.926 |
| Erstdiagnostizierte Personen absolut (in Tsd.) | 830 | 132 | 115 | 107 | 102 | 98 | 96 | 94 | 92 | 91 | 89 | 88 | 86 | 85 | 84 | 84 | 83 | 82 |
| Anteil Erstdiagnostizierte mit Schnittmengendiagnose (S) in V Vorjahren | -- | 10 % | 13 % | 14 % | 15 % | 16 % | 16 % | 17 % | 18 % | 18 % | 18 % | 19 % | 18 % | 19 % | 19 % | 19 % | 19 % | 19 % |
| Anteil Erstdiagnostizierte mit irgendeiner  F-Diagnose in V Vorjahren | -- | 74 % | 80 % | 83 % | 85 % | 87 % | 88 % | 89 % | 90 % | 91 % | 91 % | 92 % | 92 % | 93 % | 93 % | 94 % | 94 % | 94 % |
| Kennwert | Psychische Störungen (ICD-10-Kapitel V, alle Codes beginnend mit F) | | | | | | | | | | | | | | | | | |
| Erstdiagnostizierte Personen je 100.000 | 37.748 | 13.824 | 11.775 | 10.648 | 9.961 | 9.499 | 9.175 | 8.948 | 8.760 | 8.628 | 8.524 | 8.446 | 8.373 | 8.318 | 8.262 | 8.231 | 8.201 | 8.187 |
| Personen unter Risiko absolut (in Tsd.) | 83.799 | 51.855 | 45.768 | 41.172 | 37.627 | 34.700 | 32.224 | 30.116 | 28.230 | 26.644 | 25.249 | 23.987 | 22.827 | 21.801 | 20.962 | 20.129 | 19.347 | 18.630 |
| Erstdiagnostizierte Personen absolut (in Tsd.) | 31.633 | 7.168 | 5.389 | 4.384 | 3.748 | 3.296 | 2.957 | 2.695 | 2.473 | 2.299 | 2.152 | 2.026 | 1.911 | 1.813 | 1.732 | 1.657 | 1.587 | 1.525 |

* Als hinreichend versichert wurden in den jeweils insgesamt betrachteten Zeiträumen ausschließlich folgende Personen mit Versicherung bei der BARMER und inländischem Wohnort im Jahr 2022 berücksichtigt:
a) nachweislich durchgängig Versicherte, b) ggf. Neugeborene ab dem Geburtsjahr innerhalb des Beobachtungszeitraums mit nachfolgend durchgängiger Versicherung bis Ende 2022 oder Tod in 2022 sowie c) anderweitig Verstorbene im Jahr 2022 mit durchgängiger Versicherung ab Beginn des jeweils ersten Beobachtungsjahrs.
**Erläuterung:** In der 1. Ergebniszeile zu jeder Diagnosegruppe werden in Spalte 1 Prävalenzschätzer und in folgenden Spalten Inzidenzschätzer ausgewiesen. Die 2. Ergebniszeile enthält Angaben zur Population unter Risiko (Gesamtbevölkerung abzüglich der in Vorjahren diagnostizierten Personen). In der 3. Ergebniszeile werden hochgerechnete Zahlen zu Personen mit prävalenten bzw. inzidenten Diagnosen in Deutschland berichtet. Die 4. Ergebniszeile nennt Anteile dieser Personen, bei denen innerhalb von V Vorjahren Diagnosen mit psychopathologischen Schnittmengen gemäß nachfolgenden Auflistungen D, A oder S dokumentiert waren. **D**: ICD-10 F00 bis F03, F06.3, F31, F34.8, F34.9, F38, F39, F41.2, F43.2, F53, Z73; **A**: F06.4, F2, F32, F33.0 bis F33.3, F33.8, F33.9, F42, F43.1, F43.2, F45.0, F45.2, F60; **S**: F05, F06.0, F06.2, F1x.5, F1x.7, F30.2, F31.2, F31.5, F32.3, F33.3; Codes jeweils mit Berücksichtigung aller weiter differenzierten Diagnosen und mit x als Platzhalter für beliebige gültige Ziffern (zu Diagnosebezeichnungen siehe Tabelle Z10). Die 5. Ergebniszeile nennt Anteile, bei denen in Vorjahren irgendeine andere psychische Störung dokumentiert war.
**Auswertungsbasis:** Daten zu Versicherten der BARMER mit ausreichenden Beobachtungszeiten variierend von n = 4.806.658 (Versicherte mit vollständiger Vorbeobachtung über 17 Jahre, darunter n = 1.040.365 ohne Diagnose einer psychischen Störung in Vorjahren) bis n = 7.795.991 (Versicherte 2022, ohne erforderliche Vorbeobachtungszeiten); standardisiert oder hochgerechnet gemäß durchschnittlicher Bevölkerung in Deutschland 2022 nach Geschlecht, Altersgruppen und Wohnregion in Bundesländern nach Angaben des Statistischen Bundesamtes basierend auf Fortschreibungen gemäß Zensus 2011 (Angaben auf Grundlage des Zensus 2022 waren erst nach Abschluss der Analysen verfügbar; da vom Statistischen Bundesamt nur ganzzahlige Bevölkerungsangaben zu Strata bereitgestellt werden und in hier präsentierten Analysen zur Wahrung der Konsistenz stets ausdifferenzierte Angaben verwendet wurden, können berichtete Angaben zur Gesamtbevölkerung von publizierten Angaben des Statistischen Bundesamtes abweichen).

Tabelle Z10: Berücksichtigte ICD-10-Diagnoseschlüssel zu den Zieldiagnosen (Z) Depressionen, Angststörungen und Schizophrenie-Spektrum-Störungen sowie zu den jeweils zugeordneten Schnittmengendiagnosen (S)

| Gruppe | Code | Bezeichnung | Gruppe | Code | Bezeichnung |
| --- | --- | --- | --- | --- | --- |
| **D** |  | **Depressionen** | A-S | F2 | Schizophrenie, schizotype und wahnhafte Störungen |
| D-Z | F32 | Depressive Episode | A-S | F32 | Depressive Episode |
| D-Z | F33.0 | Rezidivierende depressive Störung, gegenwärtig leichte Episode | A-S | F33.0 | Rezidivierende depressive Störung, gegenwärtig leichte Episode |
| D-Z | F33.1 | Rezidivierende depressive Störung, gegenwärtig mittelgradige Episode | A-S | F33.1 | Rezidivierende depressive Störung, gegenwärtig mittelgradige Episode |
| D-Z | F33.2 | Rezidivierende depressive Störung, gegenwärtig schwere Episode ohne psychotische Symptome | A-S | F33.2 | Rezidivierende depressive Störung, gegenwärtig schwere Episode ohne psychotische Symptome |
| D-Z | F33.3 | Rezidivierende depressive Störung, gegenwärtig schwere Episode mit psychotischen Symptomen | A-S | F33.3 | Rezidivierende depressive Störung, gegenwärtig schwere Episode mit psychotischen Symptomen |
| D-Z | F33.8 | Sonstige rezidivierende depressive Störungen | A-S | F33.8 | Sonstige rezidivierende depressive Störungen |
| D-Z | F33.9 | Rezidivierende depressive Störung, nicht näher bezeichnet | A-S | F33.9 | Rezidivierende depressive Störung, nicht näher bezeichnet |
| D-Z | F34.1 | Dysthymia | A-S | F42 | Zwangsstörung |
| D-S | F00 | Demenz bei Alzheimer-Krankheit | A-S | F43.1 | Posttraumatische Belastungsstörung |
| D-S | F01 | Vaskuläre Demenz | A-S | F43.2 | Anpassungsstörungen |
| D-S | F02 | Demenz bei anderenorts klassifizierten Krankheiten | A-S | F45.0 | Somatisierungsstörung |
| D-S | F03 | Nicht näher bezeichnete Demenz | A-S | F45.2 | Hypochondrische Störung |
| D-S | F06.3 | Organische affektive Störungen | A-S | F60 | Spezifische Persönlichkeitsstörungen |
| D-S | F31 | Bipolare affektive Störung | **S** |  | **Schizophrenien inklusive schizotype und wahnhafte Störungen** |
| D-S | F34.8 | Sonstige anhaltende affektive Störungen | S-Z | F2 | Schizophrenien inklusive schizotype und wahnhafte Störungen |
| D-S | F34.9 | Anhaltende affektive Störung, nicht näher bezeichnet | S-S | F05 | Delir, nicht durch Alkohol oder andere psychotrope Substanzen bedingt |
| D-S | F38 | Andere affektive Störungen | S-S | F06.0 | Organische Halluzinose |
| D-S | F39 | Nicht näher bezeichnete affektive Störung | S-S | F06.2 | Organische wahnhafte [schizophreniforme] Störung |
| D-S | F41.2 | Angst und depressive Störung, gemischt | S-S | F1x.5 | Psychische und Verhaltensstörungen durch psychotrope Substanzen – Psychotische Störung |
| D-S | F43.2 | Anpassungsstörungen | S-S | F1x.7 | Psychische und Verhaltensstörungen durch psychotrope Substanzen – Restzustand und verzögert auftretende psychotische Störung |
| D-S | F53 | Psychische oder Verhaltensstörungen im Wochenbett, anderenorts nicht klassifiziert | S-S | F30.2 | Manie mit psychotischen Symptomen |
| D-S | Z73 | Probleme mit Bezug auf Schwierigkeiten bei der Lebensbewältigung | S-S | F31.2 | Bipolare affektive Störung, gegenwärtig manische Episode mit psychotischen Symptomen |
| **A** |  | **Angststörungen** | S-S | F31.5 | Bipolare affektive Störung, gegenwärtig schwere depressive Episode mit psychotischen Symptomen |
| A-Z | F40 | Phobische Störungen | S-S | F32.3 | Schwere depressive Episode mit psychotischen Symptomen |
| A-Z | F41 | Andere Angststörungen | S-S | F33.3 | Rezidivierende depressive Störung, gegenwärtig schwere Episode mit psychotischen Symptomen |
| A-S | F06.4 | Organische Angststörung |  |  |  |

ICD-10-GM 2022; Quelle: BfArM 2022 – Link: <https://www.bfarm.de/DE/Kodiersysteme/Services/Downloads/_node.html>
